# An interactive 3D atlas of sentinel lymph nodes in breast cancer developed using SPECT/CT

**DOI:** 10.1186/s40644-024-00738-z

**Published:** 2024-07-30

**Authors:** Josephine Situ, Poppy Buissink, Annie Mu, David K V Chung, Rob Finnegan, Thiranja P Babarenda Gamage, Tharanga D Jayathungage Don, Cameron Walker, Hayley M Reynolds

**Affiliations:** 1 Department of Engineering Science, The University of Auckland, Auckland, New Zealand; 2 Auckland Bioengineering Institute, The University of Auckland, Auckland, New Zealand; 3 Alfred Nuclear Medicine and Ultrasound, Newtown, NSW Australia; 4 Discipline of Child and Adolescent Health, Sydney Medical School, University of Sydney, Camperdown, NSW Australia; 5 Northern Sydney Cancer Centre, Royal North Shore Hospital, St Leonards, NSW Australia; 6 Institute of Medical Physics, School of Physics, The University of Sydney, Camperdown, NSW Australia; 7 Ingham Institute for Applied Medical Research, Liverpool, NSW Australia

**Keywords:** Atlas, Breast cancer, Lymph node, Sentinel lymph node, SPECT-CT

## Abstract

**Background:**

The identification and assessment of sentinel lymph nodes (SLNs) in breast cancer is important for optimised patient management. The aim of this study was to develop an interactive 3D breast SLN atlas and to perform statistical analyses of lymphatic drainage patterns and tumour prevalence.

**Methods:**

A total of 861 early-stage breast cancer patients who underwent preoperative lymphoscintigraphy and SPECT/CT were included. Lymphatic drainage and tumour prevalence statistics were computed using Bayesian inference, non-parametric bootstrapping, and regression techniques. Image registration of SPECT/CT to a reference patient CT was carried out on 350 patients, and SLN positions transformed relative to the reference CT. The reference CT was segmented to visualise bones and muscles, and SLN distributions compared with the European Society for Therapeutic Radiology and Oncology (ESTRO) clinical target volumes (CTVs). The SLN atlas and statistical analyses were integrated into a graphical user interface (GUI).

**Results:**

Direct lymphatic drainage to the axilla level I (anterior) node field was most common (77.2%), followed by the internal mammary node field (30.4%). Tumour prevalence was highest in the upper outer breast quadrant (22.9%) followed by the retroareolar region (12.8%). The 3D atlas had 765 SLNs from 335 patients, with 33.3–66.7% of axillary SLNs and 25.4% of internal mammary SLNs covered by ESTRO CTVs.

**Conclusion:**

The interactive 3D atlas effectively displays breast SLN distribution and statistics for a large patient cohort. The atlas is freely available to download and is a valuable educational resource that could be used in future to guide treatment.

**Supplementary Information:**

The online version contains supplementary material available at 10.1186/s40644-024-00738-z.

## Introduction

Breast cancer is the most commonly diagnosed cancer worldwide [1]. Without treatment, breast cancer can spread from the primary tumour site to regional lymph nodes, decreasing a patient’s chance for survival [2, 3]. Accurate identification and assessment of sentinel lymph nodes (SLNs), defined as lymph nodes which receive direct lymphatic drainage from a primary tumour site, is crucial for optimising patient management [4, 5]. The location and number of SLNs varies among breast cancer patients, however, and are best identified for each patient using lymphoscintigraphy (LS) and SPECT/CT imaging.

To better understand variations in breast lymphatic drainage and SLN distribution, previous studies have statistically analysed aggregated planar LS or 3D SPECT/CT data [6–8]. In 2004, Estourgie et al. [6] analysed 700 LS studies and computed lymphatic drainage probabilities from five breast regions. In 2012, Uren et al. [7] analysed LS and SPECT/CT data from 741 patients and reported frequencies of drainage from nine breast regions. However, neither study reported confidence intervals to quantify uncertainty in their results. For additional insights, Blumgart et al. [8] analysed data from 2304 patients imaged at the same centre as Uren et al. [7], calculating lymphatic drainage and tumour prevalence statistics with confidence intervals, and displayed results via an interactive web-based tool [8]. Despite the benefits of this tool, it was limited to a generic 2D representation of SLN location and all axillary SLNs were categorised into one node field.

Recently, several 3D atlases have been developed by mapping lymph node locations or contours from multiple breast cancer patients onto a single patient dataset [9–13]. Each 3D atlas aimed to evaluate radiation therapy clinical target volume (CTV) delineation guidelines, including the European Society for Radiotherapy and Oncology (ESTRO), and the Radiation Therapy Oncology Group (RTOG) guidelines. One atlas by Novikov et al. [13] co-registered SPECT/CT data from 254 patients to show the distribution of breast SLNs. Other atlases analysed the distribution of lymph node metastases, including one by Borm et al. [9] created using ^18^F-fluorodeoxyglucose positron emission tomography/CT (FDG-PET/CT) data from 235 patients, which they later followed with another FDG-PET/CT-derived atlas comparing metastatic and non-pathological lymph nodes in 143 patients [10]. Zhang et al. [11] developed an atlas of FDG-avid regional nodes using data from 154 patients with recurrent or advanced breast cancer. Meanwhile Beaton et al. [12] created an atlas of regional nodal recurrences using 69 PET/CT datasets. Each 3D atlas was informative, however none were presented in an interactive manner which limited their educational utility. Furthermore, lymph node distributions alongside radiation therapy CTVs were shown on 2D CT slices, making it difficult to understand their 3D spatial location.

To address these limitations, we aimed to develop an interactive 3D atlas of breast SLN distributions using LS and SPECT/CT data from a large patient cohort. We selected a new sample from the same centre as Uren et al. [7] and Blumgart et al. [8], which had enhanced SLN location information from SPECT/CT including refinement of the axillary node fields into multiple levels. We also aimed to perform statistical analysis of lymphatic drainage and tumour prevalence to complement the SLN atlas, optimising its educational and clinical utility.

## Methods

**Fig. 1:**
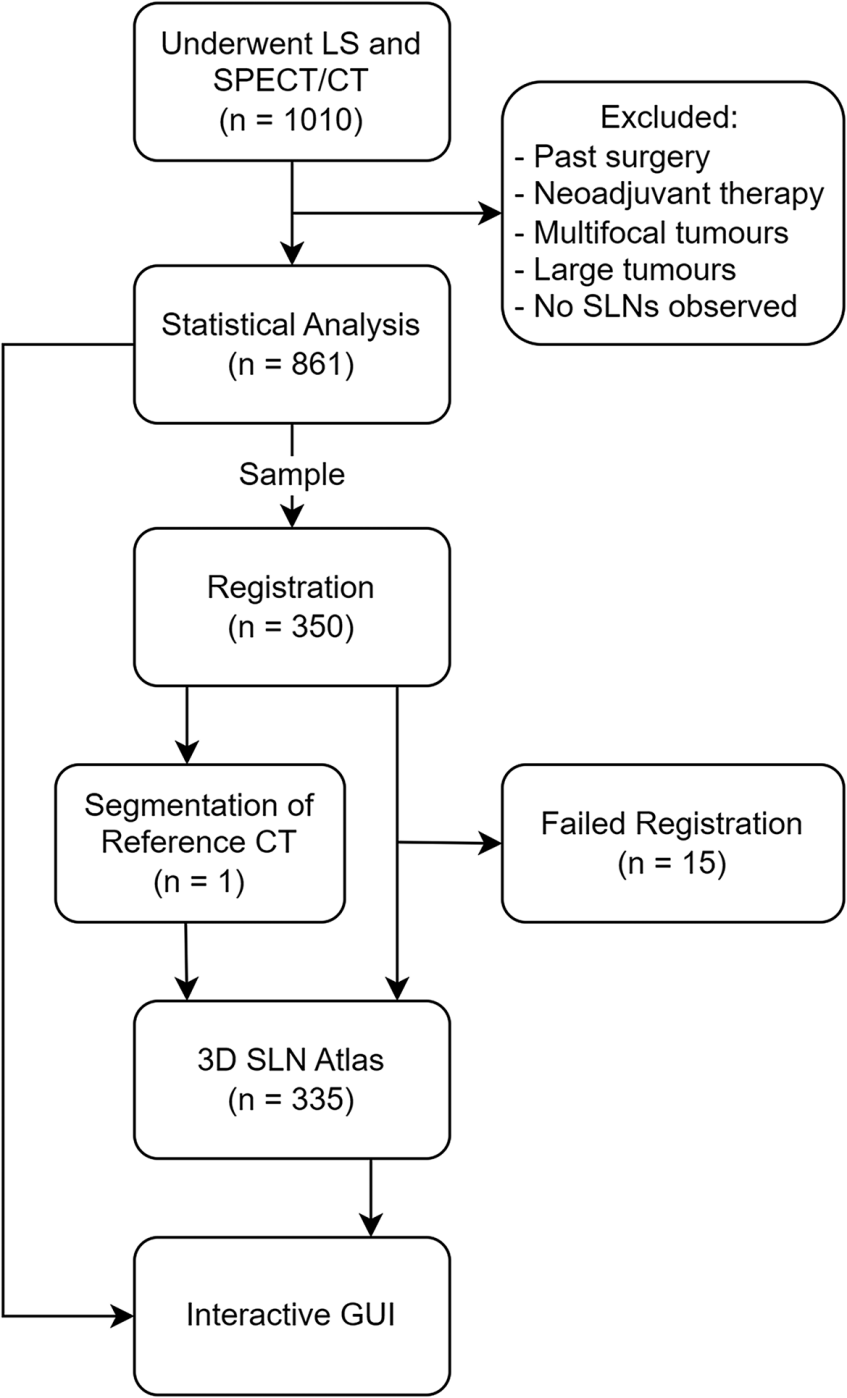
Flow chart summarising the methods and number of patients (n) involved at each step

Figure 1 summarises the workflow performed to develop the atlas, which is described in more detail in the following sections.

### Patient data

All breast cancer patients who underwent LS and SPECT/CT prior to SLN biopsy at Alfred Nuclear Medicine and Ultrasound (ANMU) in Sydney, Australia between April 2018 and December 2022 were retrospectively identified (Fig. 1). Patients who had past surgery or neoadjuvant therapy were excluded as past treatment can influence lymphatic drainage [14], and patients with multifocal disease or large tumours (diameter > 3 cm) were excluded because it was unclear which injection site was the source of the lymph flow. Details of patients in the final atlas are described in Table 1. A total of 861 patients (mean age 60.0 years) were statistically analysed, while a smaller subset of 335 patients (mean age 60.1 years) were used in the 3D SLN atlas to reduce computational power required and ensure sufficient SLN visualisation without too many overlapping SLNs. A minimum of 20 patients from each breast region were selected for the 3D SLN atlas to provide data from each region. Most patients (99.1%) had a single primary tumour visible on ultrasound, while eight patients had bilateral primary tumours and underwent LS and SPECT/CT on both breasts.

**Table 1:** Patient characteristics

Characteristic	Statistical Analysis	3D SLN Atlas
Number of patients	861	335
Number of tumours	869	336
Left tumours	452	169
Right tumours	417	167
Age, mean (range)	60.0 (25.5–92.5)	60.1 (25.5–89.3)
Female	856	335
Male	5	0

**Fig. 2:**
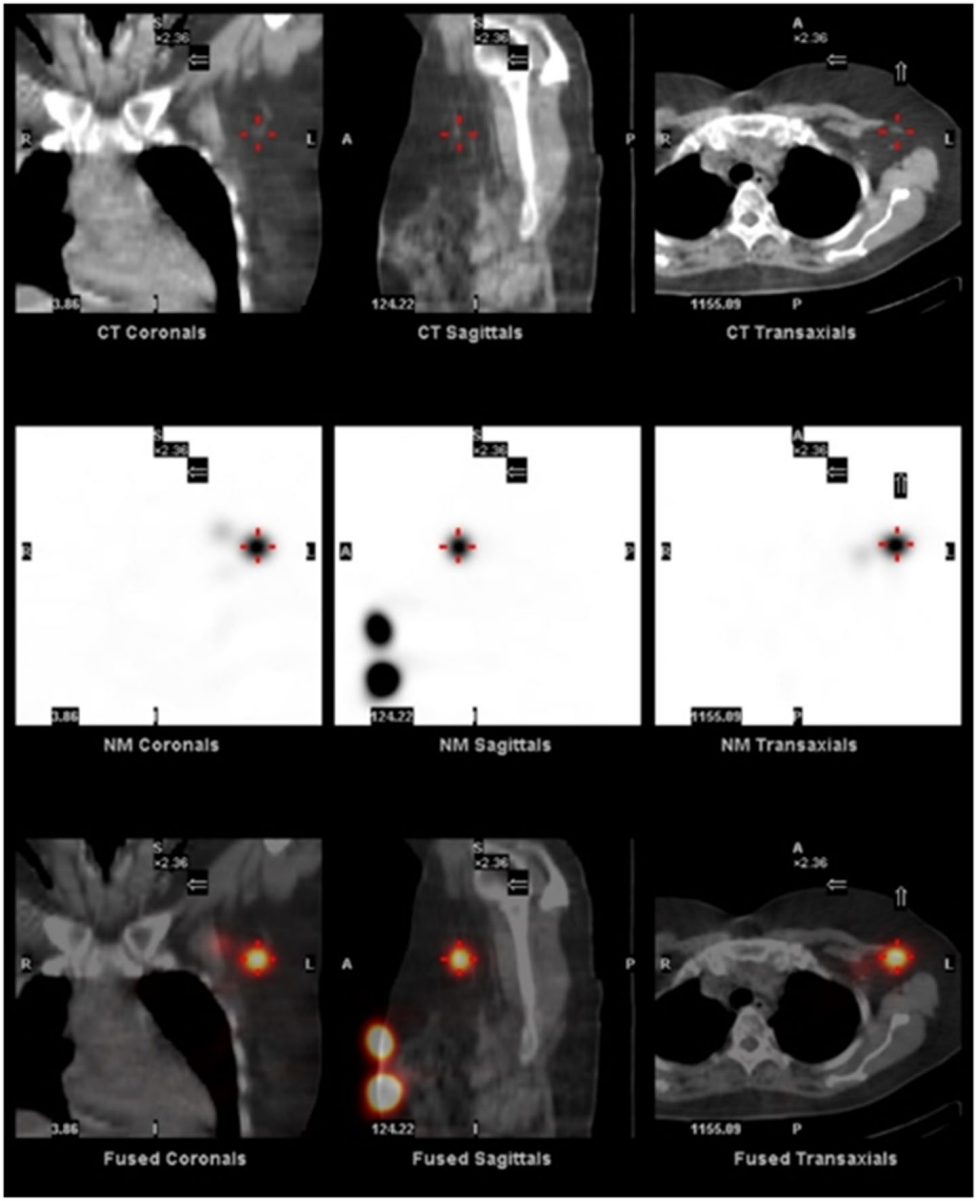
Example SPECT/CT images from a breast cancer patient showing a SLN annotated with a red cross in the axilla level I (anterior) node field

The LS and SPECT/CT procedure at the ANMU has previously been described [7]. In brief, one to four peritumoral injections of ^99m^Tc-antimony sulphide colloid were administered around the tumour under ultrasound guidance, with the needle tip less than 1 cm from the tumour edge. Following five minutes of massage around the injection site, LS was undertaken and if SLNs were observed, SPECT/CT was acquired with patients in the supine position with most patients having their arms above the head. At the time of imaging, the nuclear medicine physician annotated the SLN positions on the SPECT images (Fig. 2). The patient’s primary tumour was classified into one of 12 clockface regions, with the distance of the tumour from the nipple also recorded. If this distance was ≤ 1 cm, the tumour was reclassified as being within a 0 o’clock region. Patient SLNs were classified within one of 12 node fields, as described previously [7]: axilla level I (either anterior, central, lateral, posterior or interpectoral), axilla level II, axilla level III, internal mammary, supraclavicular, mediastinal, interval, and contralateral.

### Statistical analysis

Statistical analysis was performed using data from 861 patients (Table 1), using the R statistical package [15]. First, *drainage probabilities*, defined as the probability of SLNs being present in each node field given the primary tumour being in a particular breast region, were estimated. Second, *tumour prevalence*, defined as the probability of the primary tumour being in each breast region, given SLNs being in a certain node field, were calculated.

All data on the right side of the body was reflected to the left side of the body, as done by Blumgart et al. [8] who previously showed drainage probabilities and tumour prevalence are symmetric [16]. Data from both males and females were used, as lymphatic drainage patterns are not significantly different between sexes [16]. For patients with bilateral breast tumours (8 of 861), the lymphatic drainage from each breast was considered independently.

#### Drainage probabilities

Probability estimates were computed using Bayesian inference, non-parametric bootstrapping, and regression. As in Blumgart et al. [8], Bayesian inference was used to evaluate drainage probabilities with a uniform prior and binomial likelihood. Bayesian inference was performed in WinBUGS [17], using the *R2WinBUGS* package [18]. Bayesian posterior distributions were summarised as posterior means and 95% confidence intervals. Non-parametric bootstrapping confidence intervals were computed using 10,000 bootstrap replicates, with resampling done at a patient level. For the regression confidence intervals, a binary response variable represented the presence or absence of drainage to the selected node field, and the breast region (a categorical variable between 0 and 12) was the only predictor variable. Then, 95% Wald-based confidence intervals were calculated for the fitted values [19].

#### Tumour prevalence

To evaluate tumour prevalence using Bayesian inference, a Dirichlet prior and multinomial likelihood were used, similar to Blumgart et al. [8]. Results were summarised as posterior means and 95% confidence intervals, using WinBUGS [17]. Non-parametric bootstrapping confidence intervals were calculated as above. Regression confidence intervals for multinomial proportions were calculated using the *DescTools* package, with the default Sison and Glaz method [20].

### 3D SLN atlas development

#### Registration and segmentation

**Fig. 3:**
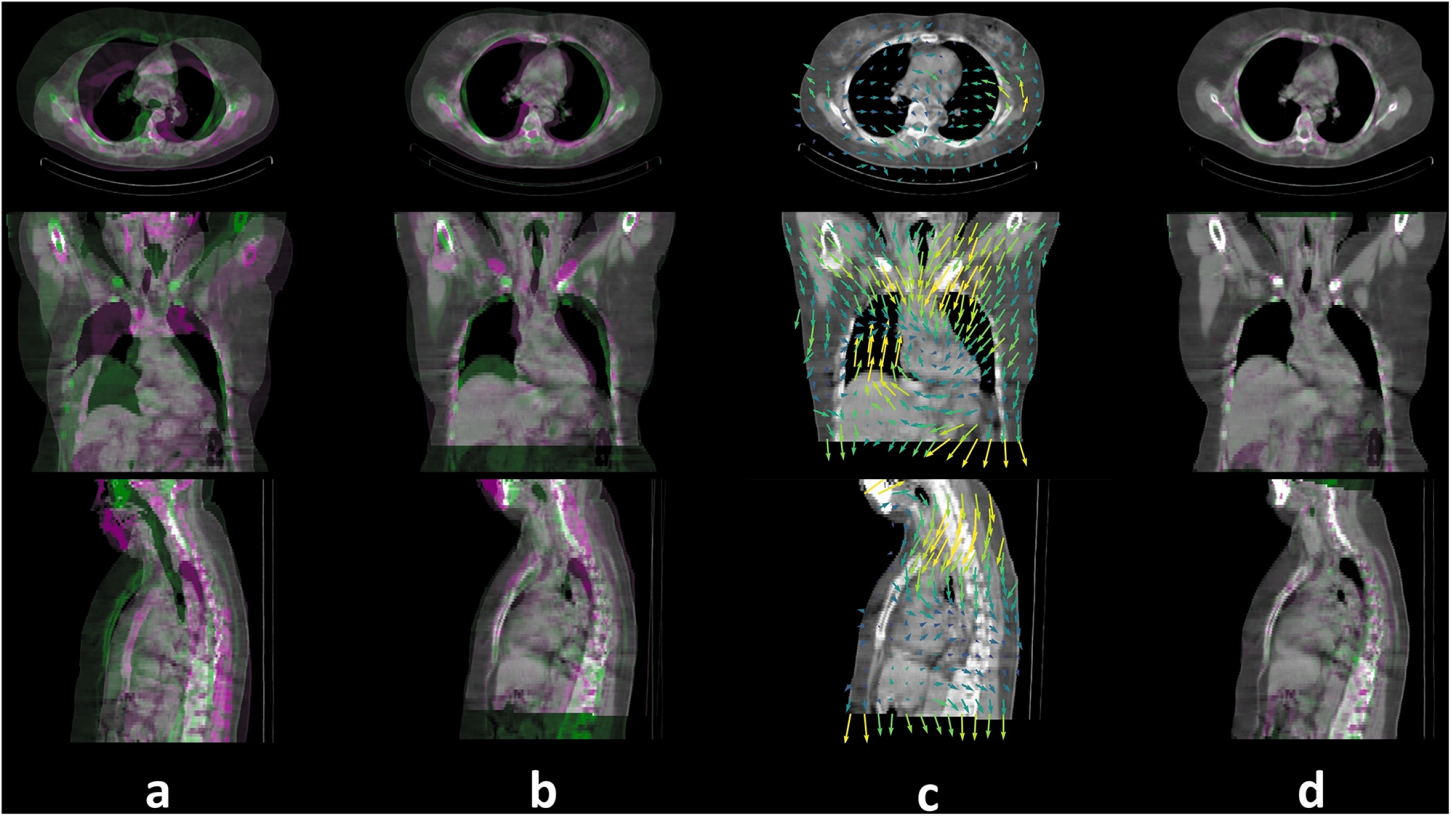
Registration workflow for an example patient, showing: **a** unregistered moving CT (purple) and reference CT images (green), **b** moving and reference CT images after linear registration, **c** deformation vector fields, **d** moving and reference CT images after deformable image registration

SPECT/CT images from 350 patients were used to construct the 3D SLN atlas (Fig. 1). All patients had SPECT/CT performed with their arms above the head, which provided consistency in patient pose when modelling inter-patient deformations during co-registration. Each patient’s CT image was registered to a reference patient CT scan, as demonstrated in Fig. 3 for an example patient. The reference CT was chosen from a random subset of 30 patients (one patient per breast tumour region plus six randomly selected patients), by identifying the patient with the median scapula length which is a predictor for body height [21] (see Table S1).

The remaining 349 CT images were registered to the reference CT with linear and deformable registration using the open-source Python library PlatiPy [22]. Linear registration gave an initial alignment and was applied using the *scaleversor* method, which performed 3D rigid transformation and anisotropic scaling [23]. Deformable registration, through the *fast symmetric forces demons* algorithm, was then used to refine the initial alignment by calculating local (voxel-wise) deformations. The 3D coordinates of each SLN identified by the nuclear medicine physician on SPECT images, were registered to the reference CT using the pre-computed linear transform and deformation vector fields.

To validate the image registration and quantify the uncertainty, CT images of the 30 patients in the previously selected sample were analysed. Thirteen skeletal landmarks (positions described in the Table S1) were manually selected by one observer in the reference CT and remaining 29 CT images, and the Euclidean distances between corresponding points were computed before and after image registration. Since estimation of the registration error was influenced by landmark annotation variability, an inter-observer study was performed. For this, three observers annotated 13 landmark points in five randomly selected patients from the 30 patient sample and the Euclidean distances between landmark points were computed.

The reference CT image was segmented using a semi-automatic approach. Bones were segmented using the 3D Slicer v5.2.2 [24] extension, *SlicerTotalSegmentator* [25], a fully automated tool based on a trained nnU-Net algorithm [26]. The sternum and anterior portions of the ribs were only partially segmented using this tool, so further manual segmentation was performed within 3D Slicer. Three muscles relevant for defining the axillary node field positions: the latissimus dorsi, pectoralis major and pectoralis minor muscles, were manually segmented on the reference patient CT image using 3D Slicer.

#### ESTRO CTV coverage

Correspondences between the ANMU SLN node fields and ESTRO CTVs are outlined in Table 2. There are no ESTRO CTVs corresponding to the ANMU mediastinal and interval SLN node fields, so these were not analysed. The ESTRO CTVs were downloaded from Offersen et al. [27] which had the left and right CTVs delineated on two different patients. Hence, the CT images from each patient were co-registered with the atlas reference CT image separately, using methods described above, and propagated to the ESTRO CTVs. The percentage SLN coverage and distances of non-covered SLN centroids from the CTVs were calculated for corresponding CTVs (Table 2) and for all CTVs. Following the criteria outlined by Zhang et al. [11], each SLN was assumed to have a diameter of 5 mm and was defined as *inside* if > 50% of its volume was within the CTV contours.

**Table 2:** ANMU SLN node fields and their corresponding ESTRO CTVs

ANMU SLN node fields	ESTRO CTV
Axilla level I (anterior, posterior, central, and lateral)	Axilla level I (L1)
Axilla level I (interpectoral)	Interpectoral nodes (INTPECT)
Axilla level II	Axilla level 2 (L2)
Axilla level III	Axilla level 3 (L3)
Supraclavicular	Lymph node level 4 (L4)
Internal mammary	Internal mammary chain (IMN and IC4^a^)

^a^ The caudal limit is generally the cranial side of the 4th rib but it can be extended by an additional intercostal space under some protocols [27].

### Interactive graphical user interface

A graphical user interface (GUI) was developed in 3D Slicer, to interactively visualise the atlas, and can be downloaded following the instructions in the supplementary information. Visualisation of the breast regions was considered important to view the associated lymphatic drainage statistics interactively. A generic schematic representation of the breast regions was created for this purpose, using segmentation tools in 3D Slicer [28].

One centrally located representative SLN from each node field was chosen to display node field names or relevant statistics. Each representative SLN was selected to ensure it was not obscured by adjacent SLNs and so its label did not overlap with other labels. The right mediastinal and left supraclavicular node fields had no SLNs from the subset of patients chosen. For these node fields, a representative SLN was manually placed in the equivalent position on the opposite side of the body.

## Results

### Patient data

The number of patients with SLNs in each node field and each breast region, after data reflection, is summarised in Table 3. The axilla level I (anterior) node field was the most common (77.2%, *n* = 671), with the second most common being the internal mammary node field (30.4%, *n* = 264). The most common primary tumour site was the 2 o’clock region, which was closest to the axilla (22.9%, *n* = 199), while the least common were the 6 o’clock and 7 o’clock regions (both 2.6%, *n* = 23).

**Table 3:** The number of patients with a primary tumour in each breast region and with SLNs in each node field

Node Field	Breast Region (Reflected to the Left Side)													# Patients with SLNs in each Node Field (% of Total)
	0 (retroareolar)	1	2	3 (lateral)	4	5	6 (inferior)	7	8	9 (medial)	10	11	12 (superior)	
Axilla level I (anterior)	87	66	164	69	44	31	20	21	19	19	42	41	48	**671 (77.2)**
Axilla level I (central)	37	19	59	29	18	11	10	5	7	6	16	14	22	**253 (29.1)**
Axilla level I (lateral)	1	3	2	1	0	0	1	0	0	0	1	1	1	**11 (1.3)**
Axilla level I (posterior)	9	6	22	12	7	7	5	1	2	0	3	7	4	**85 (9.8)**
Axilla level I (interpectoral)	5	3	5	2	1	2	0	0	0	0	0	1	2	**21 (2.4)**
Axilla level II	14	9	13	7	3	3	0	2	3	1	5	4	8	**72 (8.3)**
Axilla level III	1	0	1	1	0	0	0	0	0	0	0	0	1	**4 (0.5)**
Internal mammary	37	19	28	14	16	16	16	17	15	11	28	29	18	**264 (30.4)**
Supraclavicular	1	1	1	0	0	0	0	0	0	0	1	1	0	**5 (0.6)**
Mediastinal	1	0	0	0	0	0	1	0	0	1	0	1	0	**4 (0.5)**
Interval	13	4	16	11	4	10	5	4	1	1	3	6	5	**83 (9.6)**
Contralateral ^a^	1	0	0	0	0	0	0	0	0	0	1	0	0	**2 (0.2)**
**# Patients with Tumours in Each Breast Region** **(% of Total)**	**111 (12.8)**	**85 (9.8)**	**199 (22.9)**	**94 (10.8)**	**56 (6.4)**	**41 (4.7)**	**31 (3.6)**	**24 (2.8)**	**23 (2.6)**	**23 (2.6)**	**56 (6.4)**	**60 (6.9)**	**66 (7.6)**	**869** **(100.0)**

^a^ Both patients had contralateral drainage to axilla level I (anterior) and axilla level I (central) SLNs. One patient had contralateral drainage to an interval SLN, and the other had contralateral drainage to an internal mammary SLN.

### Statistical analysis

Table 4 presents the drainage probabilities to each node field location for all patients including point estimates and confidence intervals. For the most common axilla level I (anterior) node field, Bayesian and regression confidence intervals were the same (74.3–79.9%), while the bootstrap confidence interval was marginally higher (74.4–80.0%). The internal mammary node field had the same Bayesian and Regression drainage probabilities (27.4–33.5%), with the Bootstrap confidence interval upper bound being slightly lower (33.4%). The mean drainage probabilities and 95% confidence interval bounds were within 0.3% points across all methods.

Drainage probabilities for each separate breast region are given in Tables S2-S4. The axilla level I (anterior) node field was the most common, where all breast regions had mean drainage probabilities over 60%. The probability of SLNs in the internal mammary node field was highest from the 7 o’clock region in the lower inner breast quadrant (Bayesian mean 69.2%, bootstrap mean 71.0%, and regression mean 70.8%). When there were over 100 patients with a primary tumour in a breast region and over 20 patients with drainage to a specified node field, all three methods gave comparable results, with the mean and 95% confidence interval bounds varying by less than 1% point.

**Table 4:** Drainage probabilities to each node field for the entire breast. CI = confidence interval

Node Field	*n*	Bayesian Mean % (95% CI)	Bootstrap Mean % (95% CI)	Regression Mean % (95% CI)
Axilla level I (anterior)	671	77.2 (74.3, 79.9)	77.2 (74.4, 80.0)	77.2 (74.3, 79.9)
Axilla level I (central)	253	29.2 (26.2, 32.2)	29.1 (26.1, 32.1)	29.1 (26.2, 32.2)
Axilla level I (lateral)	11	1.4 (0.7, 2.2)	1.3 (0.6, 2.1)	1.3 (0.7, 2.3)
Axilla level I (posterior)	85	9.9 (8.0, 11.9)	9.8 (7.8, 11.8)	9.8 (8.0, 11.9)
Axilla level I (interpectoral)	21	2.5 (1.6, 3.7)	2.4 (1.5, 3.6)	2.4 (1.6, 3.7)
Axilla level II	72	8.4 (6.6, 10.3)	8.3 (6.5, 10.1)	8.3 (6.6, 10.3)
Axilla level III	4	0.6 (0.2, 1.2)	0.5 (0.1, 0.9)	0.5 (0.2, 1.2)
Internal mammary	264	30.4 (27.4, 33.5)	30.4 (27.4, 33.4)	30.4 (27.4, 33.5)
Supraclavicular	5	0.7 (0.3, 1.3)	0.6 (0.1, 1.2)	0.6 (0.2, 1.4)
Mediastinal	4	0.6 (0.2, 1.2)	0.5 (0.1, 0.9)	0.5 (0.2, 1.2)
Interval	83	9.6 (7.8, 11.7)	9.6 (7.6, 11.5)	9.6 (7.8, 11.7)
Contralateral	2	0.3 (0.1, 0.8)	0.2 (0.0, 0.6)	0.2 (0.1, 0.9)

**Table 5:** Tumour prevalence in each breast region. CI = confidence interval

Tumour Region (L)	*n*	Bayesian Mean % (95% CI)	Bootstrap Mean % (95% CI)	Regression Mean % (95% CI)
0 (retroareolar)	111	12.7 (10.6, 15.0)	12.8 (10.5, 15.1)	12.8 (9.9, 15.8)
1	85	9.7 (7.9, 11.8)	9.8 (7.8, 11.8)	9.8 (6.9, 12.8)
2	199	22.7 (20.0, 25.5)	22.9 (20.1, 25.8)	22.9 (20.0, 25.9)
3 (lateral)	94	10.8 (8.8, 12.9)	10.8 (8.8, 12.9)	10.8 (7.9, 13.9)
4	56	6.5 (4.9, 8.2)	6.5 (4.8, 8.2)	6.4 (3.6, 9.5)
5	41	4.8 (3.5, 6.3)	4.7 (3.3, 6.2)	4.7 (1.8, 7.8)
6 (inferior)	31	3.6 (2.5, 5.0)	3.6 (2.4, 4.8)	3.6 (0.7, 6.6)
7	24	2.8 (1.8, 4.0)	2.8 (1.7, 3.9)	2.8 (0.0, 5.8)
8	23	2.7 (1.8, 3.9)	2.6 (1.6, 3.8)	2.6 (0.0, 5.7)
9 (medial)	23	2.7 (1.8, 3.9)	2.6 (1.6, 3.8)	2.6 (0.0, 5.7)
10	56	6.5 (4.9, 8.2)	6.5 (4.8, 8.2)	6.4 (3.6, 9.5)
11	60	6.9 (5.3, 8.7)	6.9 (5.3, 8.7)	6.9 (4.0, 9.9)
12 (superior)	66	7.6 (5.9, 9.4)	7.6 (5.9, 9.3)	7.6 (4.7, 10.6)

Table 5 details the tumour prevalence statistics for each breast region across all patients. Tumour prevalence confidence intervals in the 2 o’clock breast region ranged from (20.0–25.5%) from Bayesian inference to (20.0–25.9%) from regression. For the retroareolar 0 o’clock breast region, Bayesian (10.6–15.0%) and bootstrap (10.5–15.1%) confidence intervals were similar. The regression confidence intervals were wider than the Bayesian or bootstrap confidence intervals, though the mean tumour prevalence was comparable. Tumour prevalence values for each node field are given in Tables S5-S7. Tumour prevalence was highest in the 2 o’clock breast region, given an SLN in the axilla level I (anterior, central, posterior, interpectoral), axilla level III, supraclavicular, and interval node fields.

### 3D SLN atlas

#### Registration and segmentation

**Fig. 4:**
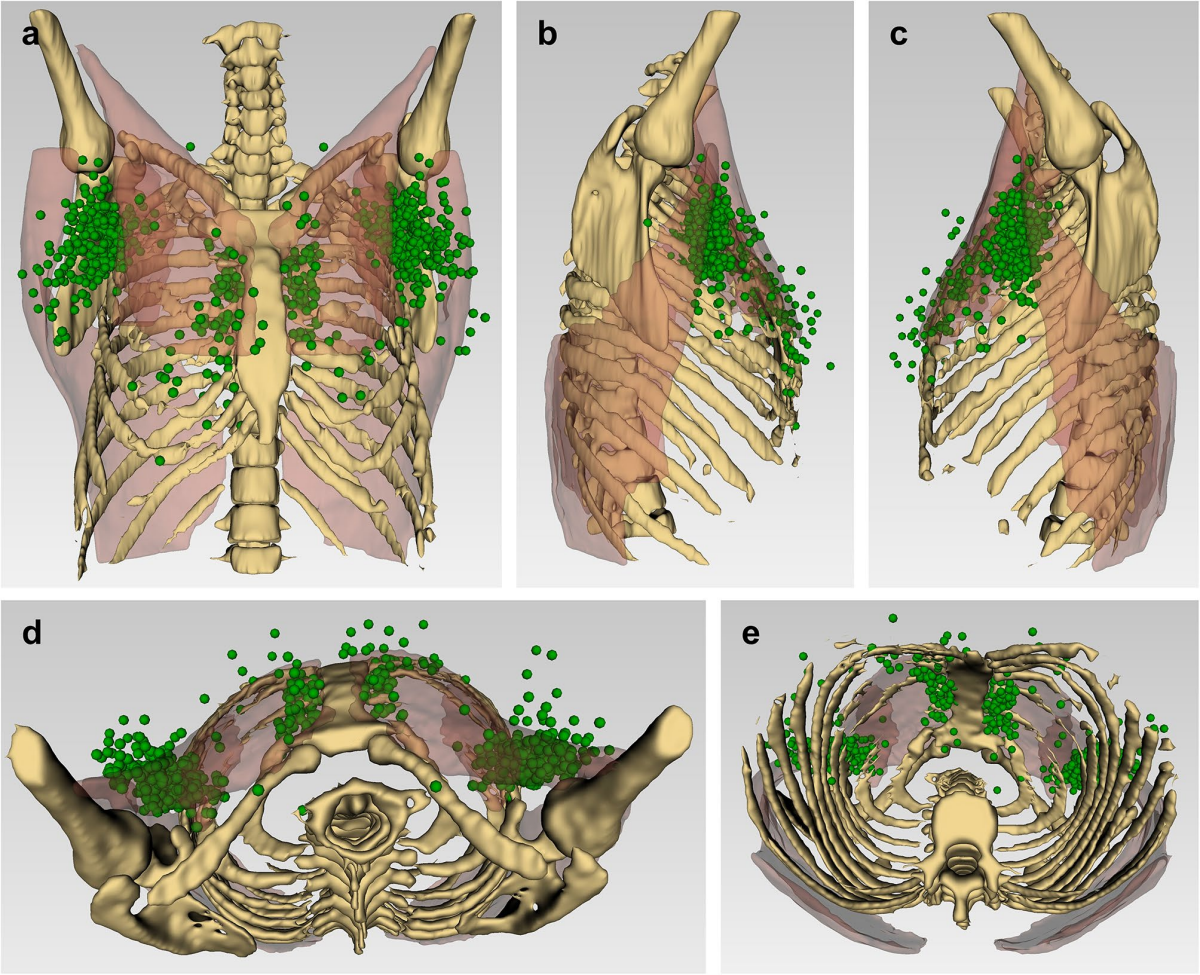
The 3D SLN atlas showing: **a** anterior, **b** right, **c** left, **d** superior, and **e** inferior views of all registered SLNs on the reference patient with segmented bones and muscles

CT images from 334 patients were successfully registered to the reference CT, while CT images from 15 patients failed to register (4.3%) due to significant anatomical differences or variations in patient positioning. The mean registration errors and inter-observer variations for each landmark are provided in Table S8. Overall, the mean registration error after linear registration was 138.7 mm (SD = 5.1 mm), which improved to 11.2 mm (SD = 4.9 mm) after deformable registration. The inter-observer variability ranged from a mean of 6.6 mm (SD = 2.7 mm) to 7.4 mm (SD = 2.8 mm). The final 3D atlas, as shown in Fig. 4, had 765 SLNs from 335 patients and an additional two manually placed SLNs in the right mediastinal and left supraclavicular node fields. Full segmentation of muscles and bones near the axilla was achieved, but the anterior ribs and full extent of the muscles were only partially segmented due to challenges with identification on CT.

#### ESTRO CTV coverage

Table 6 summarises the number of SLNs in each node field and the coverage by the corresponding ESTRO CTVs and all ESTRO CTVs. The overall percentage of axillary and internal mammary SLNs covered by all CTVs and corresponding CTVs was 36.4% and 30.8% respectively, with non-covered SLN centroids a mean distance of 5.7 mm (SD = 5.8 mm) and 5.9 (SD = 5.7 mm) respectively, from the nearest CTV border. The SLN coverage for all CTVs ranged from 33.3 to 66.7% for the axilla level I node fields, while the internal mammary node field had the lowest coverage of 25.5%.

**Table 6:** The number of SLNs in each node field in the 3D SLN atlas and associated ESTRO CTV coverage. Distance = mean ± SD of non-covered SLN centroids from the nearest CTV border

Node Field	Both Sides					Left					Right				
	*n*	Corresponding CTVs		All CTVs		*n*	Corresponding CTVs		All CTVs		*n*	Corresponding CTVs		All CTVs	
		Inside (%)	Distance (mm)	Inside (%)	Distance (mm)		Inside (%)	Distance (mm)	Inside (%)	Distance (mm)		Inside (%)	Distance (mm)	Inside (%)	Distance (mm)
Axilla level I (anterior)	354	29.7	6.6 ± 6.4	34.5	6.2 ± 6.4	175	38.3	6.0 ± 5.4	38.9	6.0 ± 5.4	179	21.2	7.0 ± 7.0	30.2	6.3 ± 7.2
Axilla level I (central)	101	35.6	7.9 ± 6.2	51.5	8.7 ± 6.7	63	34.9	8.3 ± 6.4	41.3	8.9 ± 6.5	38	36.8	7.1 ± 5.9	68.4	8.2 ± 7.6
Axilla level I (lateral)	3	33.3	2.0 ± 1.4	66.7	3.0^a^	2	50.0	3.0^a^	50.0	3.0^a^	1	0.0	1.0^a^	100.0	N/A^b^
Axilla level I (posterior)	53	37.7	4.7 ± 3.5	37.7	4.2 ± 2.8	22	40.9	3.4 ± 2.2	40.9	3.4 ± 2.2	31	35.5	5.5 ± 3.9	35.5	4.8 ± 3.1
Axilla level I (interpectoral)	6	33.3	4.0 ± 3.2	33.3	3.8 ± 3.1	2	0.0	5.4 ± 3.1	0.0	5.4 ± 3.1	4	50.0	2.7 ± 3.8	50.0	2.2 ± 3.2
Axilla level II	31	48.4	2.6 ± 1.8	64.5	1.9 ± 1.1	16	43.8	2.7 ± 1.9	75.0	1.6 ± 1.0	15	53.3	2.5 ± 1.7	53.3	2.0 ± 1.3
Axilla level III	2	0.0	4.1 ± 4.4	50.0	1.0^a^	1	0.0	1.0^a^	0.0	1.0^a^	1	0.0	7.2^a^	100.0	N/A^b^
Internal mammary	169	25.4	4.4 ± 4.3	25.4	4.4 ± 4.2	84	21.4	3.8 ± 2.9	21.4	3.7 ± 2.8	85	29.4	5.1 ± 5.3	29.4	5.1 ± 5.3
**All SLNs**	**719**	**30.8**	**5.9 ± 5.7**	**36.4**	**5.7 ± 5.8**	**365**	**34.0**	**5.5 ± 5.0**	**36.7**	**5.5 ± 5.1**	**354**	**27.6**	**6.3 ± 6.2**	**36.1**	**5.8 ± 6.4**

^a^ SD was not calculated as there was only one SLN outside of the CTVs.

^b^ No SLNs were outside of the CTVs.

### Interactive graphical user interface

**Fig. 5:**
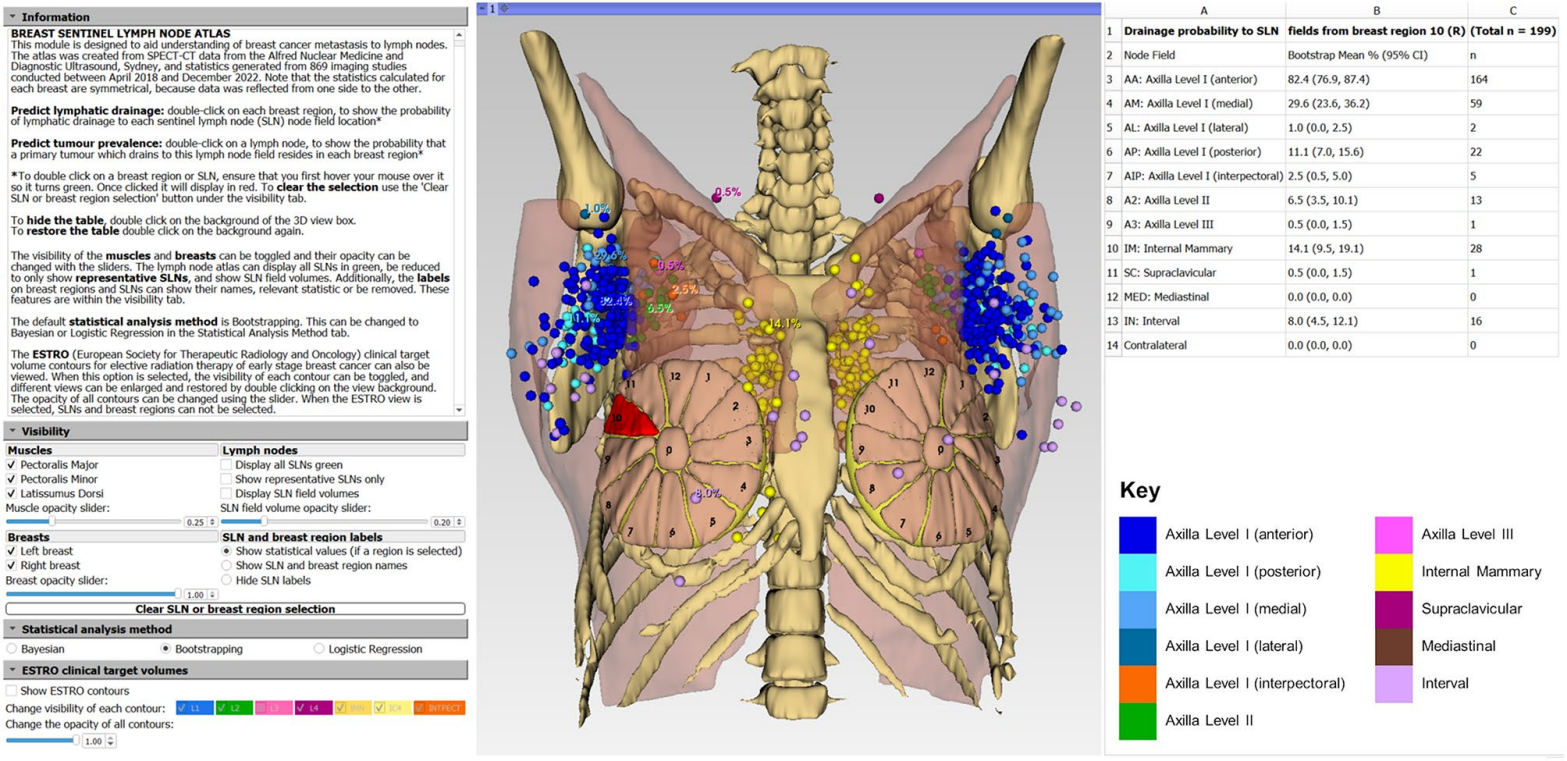
The Breast Cancer Atlas 3D Slicer module layout after selecting the right breast region 10. The SLNs are colour-coded, and drainage probabilities are shown in the table on the right. Note that selecting the corresponding left breast region (e.g., left breast region 2 here) will show the same statistics due to data reflection

**Fig. 6:**
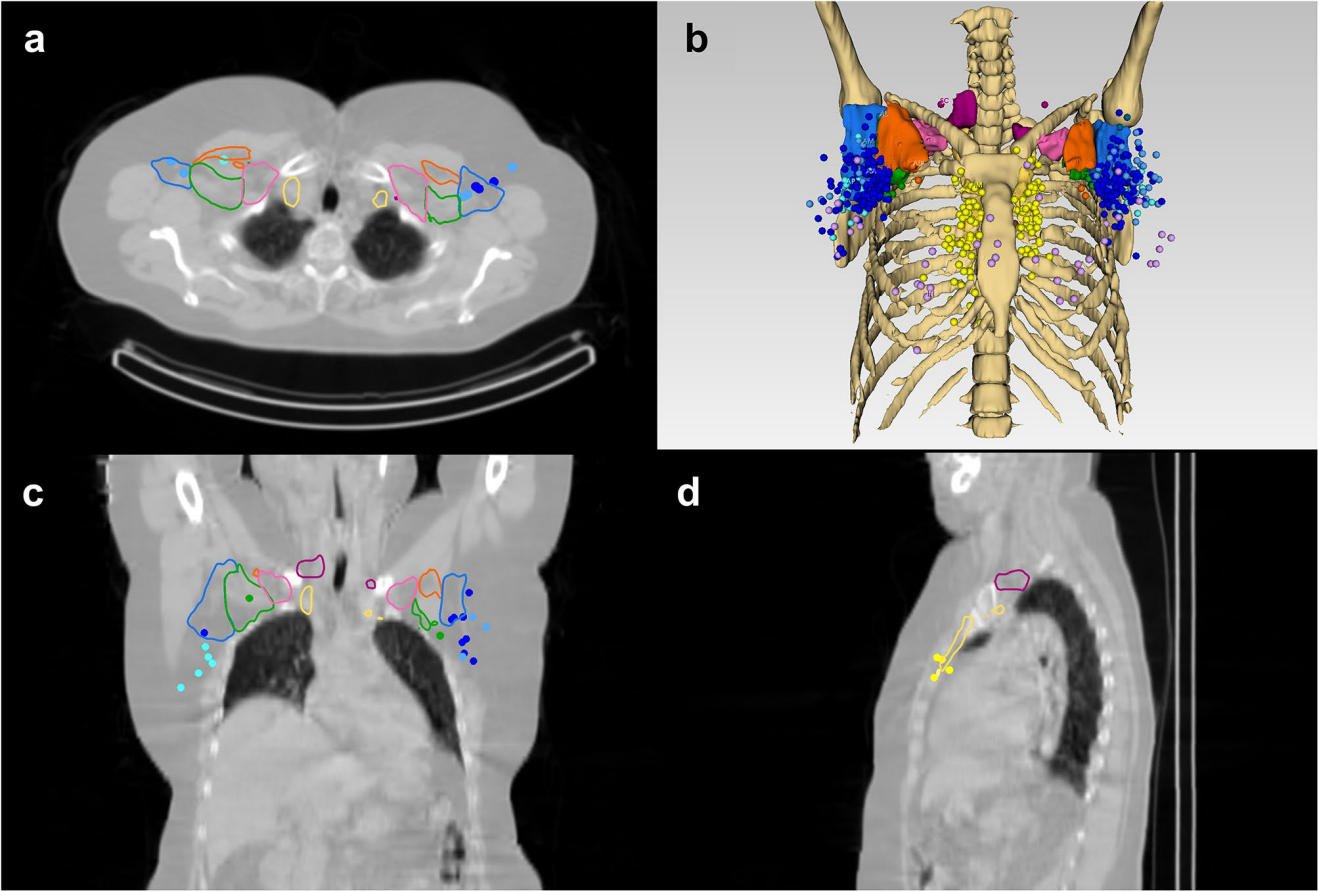
Breast Cancer Atlas 3D Slicer module layout showing **a** axial, **b** 3D perspective, **c** coronal, **d** sagittal views, when the ‘Show ESTRO contours’ checkbox is selected

The interactive GUI design is shown in Fig. 5. In the viewing window, the user can rotate, translate, and zoom in and out to achieve different perspectives. The user can interact with any breast region or SLNs by double clicking, which brings up the appropriate lymphatic drainage or tumour prevalence statistics. Within the left control panel (Fig. 5), the user can toggle the visibility and adjust the opacity of objects including the muscles and breast regions, and show SLN field volumes or representative SLNs. The co-registered ESTRO CTVs can be viewed on the reference CT in the axial, coronal, and sagittal slice views alongside the 3D rendered view (Fig. 6).

## Discussion

To our knowledge, this is the first interactive anatomically based 3D atlas of SLNs that drain the breast which combines the 3D anatomical distribution of SLN positions with comprehensive statistical analyses. The study has several strengths and advantages, including the largest number of patient images used to create an atlas, and the precise anatomical localisation of SLNs using SPECT/CT data. No other 3D anatomical breast SLN atlases [9–13] have incorporated multiple statistical analyses, and previous studies have either not reported confidence intervals to quantify uncertainty [6, 7], or used only Bayesian inference [8]. The use of more detailed classifications of node field locations compared to Blumgart et al. [8] and Estourgie et al. [6] is an additional advantage.

The drainage probabilities reported here are comparable with previous studies [6–8], with axillary and internal mammary drainage being the most common while SLNs tend to be in node fields closest to the primary tumour [8]. Similarly, drainage to the internal mammary node field were higher from primary tumours in the 7 and 8 o’clock regions, which is consistent with the lower inner quadrant region drainage results in Estourgie et al. [6], Uren et al. [7], and Blumgart et al. [8]. Most tumours were in the 2 o’clock region (closest to the axilla), consistent with the upper outer breast quadrant being most common in Estourgie et al. [6], Uren et al. [7], and Blumgart et al. [8]. The retroareolar 0 o’clock breast region was the second most common tumour location, with a higher prevalence (12.8%; bootstrapping 95% CI: 10.5–15.1%) reported in this study compared to the results of Estourgie et al. [6] (8.7%), Uren et al. [7] (4.2%), and Blumgart et al. [8] (4.0%), which could be due to differences in region size between studies.

The co-registration of patient CT images to a reference CT allowed SLN positions from a large representative sample of patients to be aligned to a common patient geometry. The registration process was successful for over 95% of CT images, and CT images for which registration failed typically had large anatomical variations compared to the reference CT. Computation of registration error provided quantitative metrics of the accuracy and precision of SLN positions, not provided by other breast atlases [9–13]. The estimated registration error (mean 11.2 mm) was slightly larger than the inter-observer variation (mean 7.4 mm for Observers 2 and 3) and the reference CT slice thickness (4.4 mm). This indicates that misalignments between the reference and moving images persisted after registration. However, as the registration error was only slightly larger than the inter-observer variation, the SLN positions were regarded suitable for this work. Registration and segmentation accuracy is primarily limited by the quality of the CT images, which are subject to motion-related blurring, poor soft-tissue contrast, and image artefacts. Anatomical differences between the reference and other patients may not be accounted for by image registration alone.

Although the purpose of the atlas is not to guide radiotherapy, comparisons with ESTRO CTVs were performed. Coverage ranged from 33.3 to 66.7% for the axilla level I node fields, which was lower than Novikov et al. [13], who found 82.7% coverage for the entire axilla level I node field. However, Novikov et al. [13], calculated coverage per patient with less restrictive criteria (> 10% SLN overlap was considered covered, compared to > 50% in this study). In addition to criteria differences, discordant results may be due to ESTRO CTV distortion during the registration process, demonstrated by the asymmetrical CTVs in Fig. 6. Non-covered SLNs were a mean distance of 5.7 mm away from the nearest CTV boundary, which may be covered when margins are added for planning purposes [27]. Future work to analyse discrepancies could involve manual delineation of ESTRO CTVs on the reference CT by radiation oncologists, as performed in other studies [11–13]. An additional limitation to our work is the low-dose reference CT slice thickness of 4.4 mm, which was larger than the 2–3 mm recommended for CTV delineation by Offerson et al. [27].

There are several limitations to this study. The 3D SLN atlas contains data from female patients only, while the statistical analysis has a very small number of male patients, and hence neither may be representative of a male cohort. The reference CT image was a low dose CT scan with relatively thick slices, which meant it was not possible to visualise and segment blood vessels which are important for SLN identification. Therefore, future work could involve using a higher quality diagnostic CT scan as the reference image which could enable visualisation of relevant blood vessels. This would also improve registration and segmentation accuracy.

## Conclusions

This study presents the first interactive 3D atlas of breast SLNs developed using SPECT/CT data with integrated statistical results. The 3D atlas enhances our understanding of breast lymphatic drainage patterns and tumour prevalence from a large patient cohort and provides a valuable resource for medical education which has potential to aid breast cancer treatment planning.

### Electronic supplementary material

Below is the link to the electronic supplementary material.

Supplementary Material 1

## Data Availability

The interactive SLN atlas is freely available for download following the instructions in the supplementary information. The data used to create the atlas are available from the corresponding author on reasonable request.

## Abbreviations

ANMU: Alfred Nuclear Medicine and Ultrasound
CI: Confidence interval
CT: Computed tomography
CTV: Clinical target volume
ESTRO: European Society for Therapeutic Radiology and Oncology
FDG-PET: ^18^F-fluorodeoxyglucose positron emission tomography
GUI: Graphical user interface
LS: Lymphoscintigraphy
RTOG: Radiation Therapy Oncology Group
SLN: Sentinel lymph node
SPECT: Single photon emission computed tomography
