# Supplementary material for: An interactive 3D atlas of sentinel lymph nodes in breast cancer developed using SPECT/CT
Source: Cancer Imaging. 2024 Jul 30;24:97. doi: 10.1186/s40644-024-00738-z (PMC11289966; doi:10.1186/s40644-024-00738-z)
Supplement: Supplementary file 1 — Supplementary Material 1 [file 40644_2024_738_MOESM1_ESM.pdf]

# Supplementary Material

## Interactive GUI Installation Instructions

The interactive GUI is a 3D Slicer extension that can be installed following the steps below.

1. Go to https://github.com/jsit433/BreastCancerAtlasExtension and download the folder 'BreastCancerAtlasExtension' by selecting the green 'Code' button > Download ZIP. Unzip the folder and then save this in any accessible location.

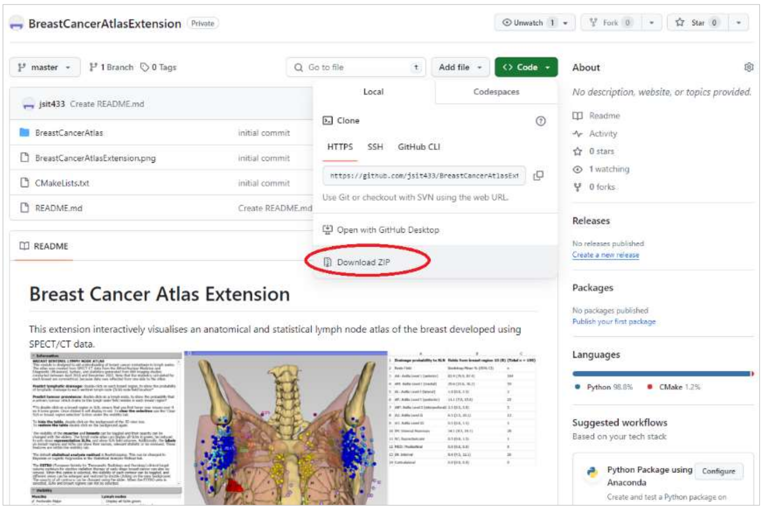


2. Download and install 3D Slicer version 5.2.2 (https://download.slicer.org/?version=5.2.2).

3. Start 3D Slicer.

4. Under the module selection tab labelled ‘Welcome to Slicer’ select Developer Tools > Extension Wizard.

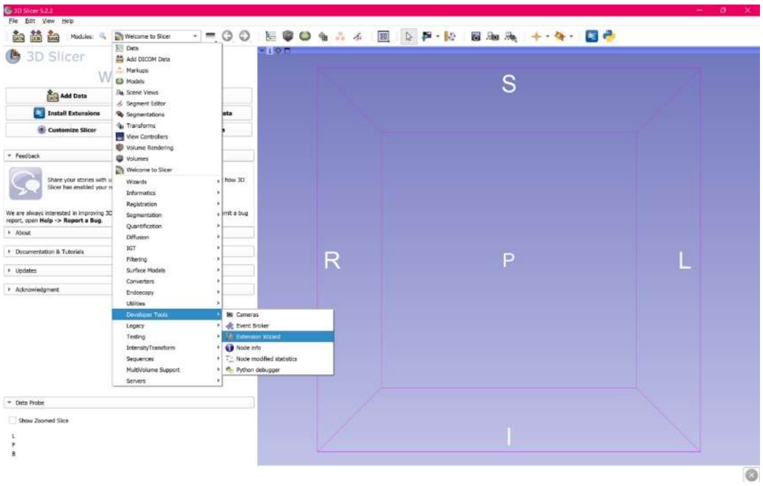

5. Click ‘Select Extension’.

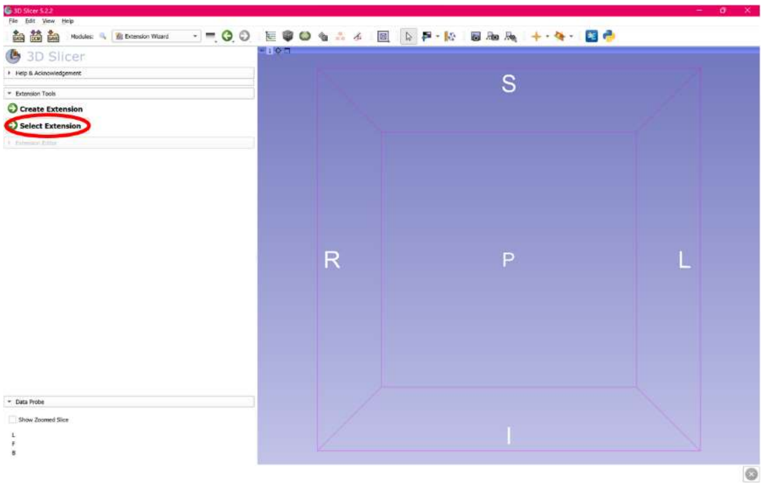


6. Locate the unzipped BreastCancerAtlasExtension-master folder from where you saved it earlier. Ensure the folder you select contains a 'BreastCancerAtlas' folder (you may have to double click twice on 'BreastCancerAtlasExtension-master'). Deselect ‘Enable developer mode’. If it asks you to install command line developer tools please do so.

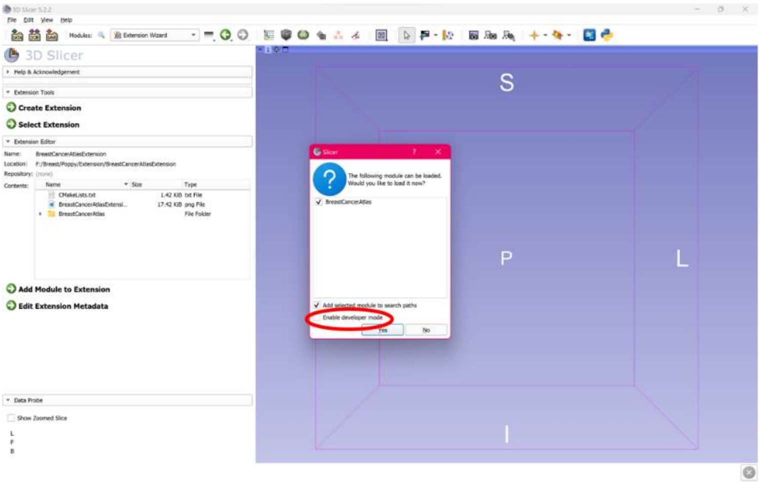


7. After pressing 'Yes', close Slicer and then reopen it. The module should load when Slicer is reopened.

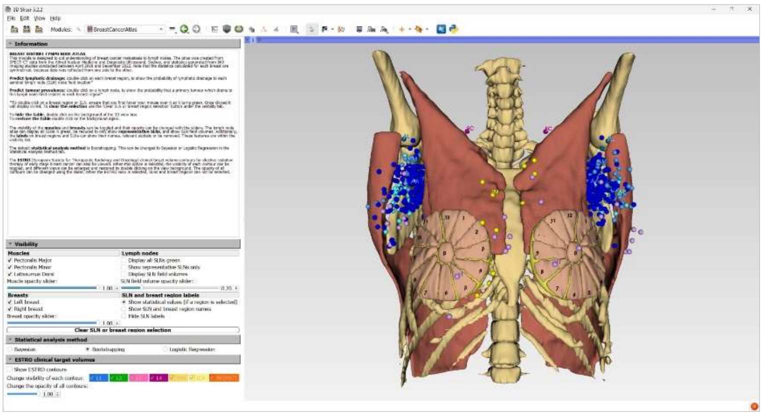

8. If the information and options in the left control panel do not immediately appear, under the module selection tab labelled 'Welcome to Slicer' select Custom Modules > BreastCancerAtlas

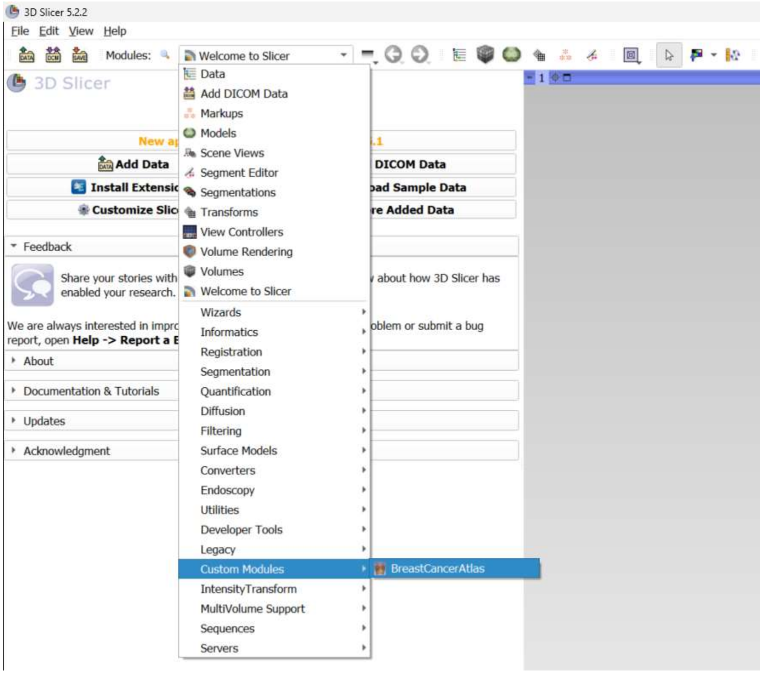

**Skeletal Landmark Location Descriptions**

**Table S1** Skeletal landmark location descriptions

| Landmark | Position Description |
|---|---|
| left scapula | intersection between left scapula and left clavicle |
| right scapula | intersection between right scapula and right clavicle |
| left humerus | the inferior border of left humerus head groove |
| right humerus | the inferior border of right humerus head groove |
| left clavicle | intersection between left clavicle and sternum |
| right clavicle | intersection between right clavicle and sternum |
| sternum | the suprasternal notch of the sternum |
| left rib 2 | the left inferior point of the sternum-second rib joint |
| right rib 2 | the right inferior point of the sternum-second rib joint |
| left rib 3 | the left inferior point of the sternum-third rib joint |
| right rib 3 | the right inferior point of the sternum-third rib joint |
| left inferior scapula | the most inferior point located on the left scapula |
| right inferior scapula | the most inferior point on the right scapula |

## Drainage Probabilities

**Table S2** Bayesian drainage probabilities to each node field from each tumour region, reported as mean % (95% CI)

| Tumour Region (L breast) | Node Field | | | | | | | | | | | |
|---|---|---|---|---|---|---|---|---|---|---|---|---|
| | Axilla Level I (anterior) | Axilla Level I (medial) | Axilla Level I (lateral) | Axilla Level I (posterior) | Axilla Level I (inter-pectoral) | Axilla Level II | Axilla Level III | Internal Mammary | Supra-clavicular | Mediastinal | Interval | Contra-lateral |
| 0 (central) | 77.9 (69.8, 85.0) | 33.6 (25.3, 42.6) | 1.8 (0.2, 4.9) | 8.9 (4.4, 14.7) | 5.3 (2.0, 10.1) | 13.3 (7.7, 20.1) | 1.8 (0.2, 4.9) | 33.6 (25.3, 42.6) | 1.8 (0.2, 4.9) | 1.8 (0.2, 4.9) | 12.4 (7.0, 19.0) | 1.8 (0.2, 4.9) |
| 1 | 77.0 (67.7, 85.2) | 23.0 (14.8, 32.3) | 4.6 (1.3, 9.8) | 8.0 (3.3, 14.6) | 4.6 (1.3, 9.8) | 11.5 (5.7, 19.0) | 1.2 (0.0, 4.2) | 23.0 (14.8, 32.3) | 2.3 (0.3, 6.3) | 1.1 (0.0, 4.2) | 5.8 (1.9, 11.5) | 1.2 (0.0, 4.2) |
| 2 | 82.1 (76.5, 87.1) | 29.8 (23.7, 36.3) | 1.5 (0.3, 3.6) | 11.4 (7.4, 16.2) | 3.0 (1.1, 5.7) | 7.0 (3.9, 10.9) | 1.0 (0.1, 2.8) | 14.4 (9.9, 19.6) | 1.0 (0.1, 2.8) | 0.5 (0.0, 1.8) | 8.5 (5.0, 12.7) | 0.5 (0.0, 1.8) |
| 3 (lateral) | 72.9 (63.6, 81.3) | 31.3 (22.4, 40.8) | 2.1 (0.3, 5.7) | 13.5 (7.5, 21.0) | 3.1 (0.7, 7.4) | 8.3 (3.7, 14.6) | 2.1 (0.3, 5.7) | 15.6 (9.1, 23.5) | 1.0 (0.0, 3.8) | 1.0 (0.0, 3.8) | 12.5 (6.7, 19.8) | 1.0 (0.0, 3.8) |
| 4 | 77.6 (66.1, 87.3) | 32.8 (21.4, 45.2) | 1.7 (0.0, 6.3) | 13.8 (6.3, 23.7) | 3.4 (0.4, 9.4) | 6.9 (1.9, 14.6) | 1.7 (0.0, 6.3) | 29.3 (18.4, 41.5) | 1.7 (0.0, 6.3) | 1.7 (0.0, 6.2) | 8.6 (2.9, 17.0) | 1.7 (0.0, 6.3) |
| 5 | 74.4 (60.6, 86.1) | 27.9 (15.7, 42.0) | 2.3 (0.1, 8.4) | 18.6 (8.6, 31.3) | 7.0 (1.5, 16.1) | 9.3 (2.7, 19.5) | 2.3 (0.1, 8.4) | 39.5 (25.6, 54.4) | 2.3 (0.1, 8.4) | 2.3 (0.1, 8.4) | 25.6 (13.9, 39.5) | 2.3 (0.1, 8.4) |
| 6 (inferior) | 63.6 (46.8, 78.8) | 33.3 (18.6, 50.0) | 6.1 (0.8, 16.2) | 18.2 (7.2, 32.8) | 3.0 (0.1, 10.9) | 3.0 (0.1, 10.9) | 3.0 (0.1, 10.9) | 51.5 (34.7, 68.1) | 3.0 (0.1, 10.9) | 6.1 (0.8, 16.2) | 18.2 (7.2, 32.8) | 3.0 (0.1, 10.9) |
| 7 | 84.6 (68.8, 95.5) | 23.1 (9.3, 40.7) | 3.8 (0.1, 13.7) | 7.7 (1.0, 20.3) | 3.8 (0.1, 13.7) | 11.5 (2.5, 26.0) | 3.8 (0.1, 13.7) | 69.2 (50.6, 85.1) | 3.8 (0.1, 13.7) | 3.8 (0.1, 13.7) | 19.2 (6.8, 36.1) | 3.8 (0.1, 13.7) |
| 8 | 80 (62.6, 92.9) | 32 (15.7, 51.2) | 4 (0.1, 14.2) | 12 (2.7, 27.0) | 4 (0.1, 14.3) | 16 (4.7, 32.4) | 4 (0.1, 14.2) | 64 (44.7, 81.2) | 4 (0.1, 14.3) | 4 (0.1, 14.2) | 8 (1.0, 21.1) | 4 (0.1, 14.2) |
| 9 (medial) | 80 (62.7, 92.8) | 28 (12.6, 46.7) | 4 (0.1, 14.2) | 4 (0.1, 14.3) | 4 (0.1, 14.2) | 8 (1.0, 21.2) | 4 (0.1, 14.2) | 48 (29.1, 67.2) | 4 (0.1, 14.3) | 8 (1.0, 21.1) | 8 (1.0, 21.1) | 4 (0.1, 14.2) |
| 10 | 74.1 (62.2, 84.5) | 29.3 (18.4, 41.5) | 3.4 (0.4, 9.4) | 6.9 (1.9, 14.6) | 1.7 (0.0, 6.3) | 10.4 (4.0, 19.3) | 1.7 (0.0, 6.3) | 50.0 (37.3, 62.7) | 3.4 (0.4, 9.4) | 1.7 (0.0, 6.3) | 6.9 (1.9, 14.6) | 3.4 (0.4, 9.4) |
| 11 | 67.8 (55.7, 78.7) | 24.2 (14.5, 35.5) | 3.2 (0.4, 8.8) | 12.9 (5.8, 22.2) | 3.2 (0.4, 8.8) | 8.1 (2.7, 16.0) | 1.6 (0.0, 5.9) | 48.4 (36.1, 60.8) | 3.2 (0.4, 8.8) | 3.2 (0.4, 8.8) | 11.3 (4.7, 20.2) | 1.6 (0.0, 5.9) |
| 12 (superior) | 72.1 (60.9, 82.0) | 33.8 (23.2, 45.4) | 2.9 (0.4, 8.0) | 7.4 (2.5, 14.6) | 4.4 (0.9, 10.4) | 13.2 (6.3, 22.2) | 2.9 (0.4, 8.0) | 27.9 (18.0, 39.1) | 1.5 (0.0, 5.4) | 1.5 (0.0, 5.4) | 8.8 (3.4, 16.5) | 1.5 (0.0, 5.4) |
| All | 77.2 (74.3, 79.9) | 29.2 (26.2, 32.2) | 1.4 (0.7, 2.2) | 9.9 (8.0, 11.9) | 2.5 (1.6, 3.7) | 8.4 (6.6, 10.3) | 0.6 (0.2, 1.2) | 30.4 (27.4, 33.5) | 0.7 (0.3, 1.3) | 0.6 (0.2, 1.2) | 9.6 (7.8, 11.7) | 0.3 (0.1, 0.8) |

**Table S3** Bootstrap drainage probabilities to each node field from each tumour region, reported as mean % (95% CI)

| Tumour Region (L breast) | Node Field | | | | | | | | | | | |
|---|---|---|---|---|---|---|---|---|---|---|---|---|
| | Axilla Level I (anterior) | Axilla Level I (medial) | Axilla Level I (lateral) | Axilla Level I (posterior) | Axilla Level I (inter-pectoral) | Axilla Level II | Axilla Level III | Internal Mammary | Supra-clavicular | Mediastinal | Interval | Contra-lateral |
| **0 (central)** | 78.4 (70.3, 85.6) | 33.3 (24.3, 42.3) | 0.9 (0.0, 2.7) | 8.1 (3.6, 13.5) | 4.5 (0.9, 9.0) | 12.6 (7.2, 18.9) | 0.9 (0.0, 2.7) | 33.3 (24.3, 42.3) | 0.9 (0.0, 2.7) | 0.9 (0.0, 2.7) | 11.7 (6.3, 18.0) | 0.9 (0.0, 2.7) |
| **1** | 77.6 (68.2, 85.9) | 22.4 (14.1, 31.8) | 3.5 (0.0, 8.2) | 7.1 (2.4, 12.9) | 3.5 (0.0, 8.2) | 10.6 (4.7, 17.6) | 0.0 (0.0, 0.0) | 22.3 (14.1, 31.8) | 1.2 (0.0, 3.5) | 0.0 (0.0, 0.0) | 4.7 (1.2, 9.4) | 0.0 (0.0, 0.0) |
| **2** | 82.4 (76.9, 87.4) | 29.6 (23.6, 36.2) | 1.0 (0.0, 2.5) | 11.1 (7.0, 15.6) | 2.5 (0.5, 5.0) | 6.5 (3.5, 10.1) | 0.5 (0.0, 1.5) | 14.1 (9.5, 19.1) | 0.5 (0.0, 1.5) | 0.0 (0.0, 0.0) | 8.0 (4.5, 12.1) | 0.0 (0.0, 0.0) |
| **3 (lateral)** | 73.4 (63.8, 81.9) | 30.9 (21.3, 40.4) | 1.1 (0.0, 3.2) | 12.7 (6.4, 20.2) | 2.1 (0.0, 5.3) | 7.5 (3.2, 12.8) | 1.1 (0.0, 3.2) | 14.9 (8.5, 22.3) | 0.0 (0.0, 0.0) | 0.0 (0.0, 0.0) | 11.7 (5.3, 18.1) | 0.0 (0.0, 0.0) |
| **4** | 78.6 (67.9, 89.3) | 32.2 (19.6, 44.6) | 0.0 (0.0, 0.0) | 12.5 (5.4, 21.4) | 1.8 (0.0, 5.4) | 5.4 (0.0, 12.5) | 0.0 (0.0, 0.0) | 28.6 (17.9, 41.1) | 0.0 (0.0, 0.0) | 0.0 (0.0, 0.0) | 7.1 (1.8, 14.3) | 0.0 (0.0, 0.0) |
| **5** | 75.6 (61.0, 87.8) | 26.8 (14.6, 41.5) | 0.0 (0.0, 0.0) | 17.1 (7.3, 29.3) | 4.9 (0.0, 12.2) | 7.3 (0.0, 17.1) | 0.0 (0.0, 0.0) | 39.0 (24.4, 53.7) | 0.0 (0.0, 0.0) | 0.0 (0.0, 0.0) | 24.3 (12.2, 39.0) | 0.0 (0.0, 0.0) |
| **6 (inferior)** | 64.5 (48.4, 80.6) | 32.3 (16.1, 48.4) | 3.2 (0.0, 9.7) | 16.1 (3.2, 29.0) | 0.0 (0.0, 0.0) | 0.0 (0.0, 0.0) | 0.0 (0.0, 0.0) | 51.6 (35.5, 67.7) | 0.0 (0.0, 0.0) | 3.2 (0.0, 9.7) | 16.1 (3.2, 29.0) | 0.0 (0.0, 0.0) |
| **7** | 87.5 (75.0, 100.0) | 20.8 (4.2, 37.5) | 0.0 (0.0, 0.0) | 4.2 (0.0, 12.5) | 0.0 (0.0, 0.0) | 8.3 (0.0, 20.8) | 0.0 (0.0, 0.0) | 70.8 (50.0, 87.5) | 0.0 (0.0, 0.0) | 0.0 (0.0, 0.0) | 16.7 (4.2, 33.3) | 0.0 (0.0, 0.0) |
| **8** | 82.6 (65.2, 95.7) | 30.5 (13.0, 47.8) | 0.0 (0.0, 0.0) | 8.7 (0.0, 21.7) | 0.0 (0.0, 0.0) | 13.1 (0.0, 26.1) | 0.0 (0.0, 0.0) | 65.2 (43.5, 82.6) | 0.0 (0.0, 0.0) | 0.0 (0.0, 0.0) | 4.3 (0.0, 13.0) | 0.0 (0.0, 0.0) |
| **9 (medial)** | 82.6 (65.2, 95.7) | 26.1 (8.7, 43.5) | 0.0 (0.0, 0.0) | 0.0 (0.0, 0.0) | 0.0 (0.0, 0.0) | 4.4 (0.0, 13.0) | 0.0 (0.0, 0.0) | 47.7 (26.1, 69.6) | 0.0 (0.0, 0.0) | 4.4 (0.0, 13.0) | 4.4 (0.0, 13.0) | 0.0 (0.0, 0.0) |
| **10** | 75.0 (62.5, 85.7) | 28.6 (17.9, 41.1) | 1.8 (0.0, 5.4) | 5.4 (0.0, 12.5) | 0.0 (0.0, 0.0) | 9.0 (1.8, 17.9) | 0.0 (0.0, 0.0) | 50.0 (37.5, 62.5) | 1.8 (0.0, 5.4) | 0.0 (0.0, 0.0) | 5.4 (0.0, 12.5) | 1.8 (0.0, 5.4) |
| **11** | 68.3 (56.7, 80.0) | 23.3 (13.3, 35.0) | 1.7 (0.0, 5.0) | 11.7 (5.0, 20.0) | 1.7 (0.0, 5.0) | 6.7 (1.7, 13.3) | 0.0 (0.0, 0.0) | 48.3 (35.0, 61.7) | 1.7 (0.0, 5.0) | 1.7 (0.0, 5.0) | 10.0 (3.3, 18.3) | 0.0 (0.0, 0.0) |
| **12 (superior)** | 72.7 (62.1, 83.3) | 33.3 (22.7, 45.5) | 1.5 (0.0, 4.5) | 6.1 (1.5, 12.1) | 3.0 (0.0, 7.6) | 12.1 (4.5, 19.7) | 1.5 (0.0, 4.5) | 27.3 (16.7, 37.9) | 0.0 (0.0, 0.0) | 0.0 (0.0, 0.0) | 7.6 (1.5, 15.2) | 0.0 (0.0, 0.0) |
| **All** | 77.2 (74.5, 80.0) | 29.1 (26.1, 32.1) | 1.3 (0.6, 2.1) | 9.8 (7.8, 11.9) | 2.4 (1.5, 3.5) | 8.3 (6.4, 10.1) | 0.5 (0.1, 0.9) | 30.4 (27.4, 33.5) | 0.6 (0.1, 1.2) | 0.5 (0.1, 0.9) | 9.6 (7.6, 11.5) | 0.2 (0.0, 0.6) |

**Table S4** Regression drainage probabilities to each node field from each tumour region, reported as mean % (95% CI). Note: 'nan' indicates that a confidence interval could not be calculated

| Tumour Region (L breast) | Node Field | | | | | | | | | | | |
|---|---|---|---|---|---|---|---|---|---|---|---|---|
| | **Axilla Level I (anterior)** | **Axilla Level I (medial)** | **Axilla Level I (lateral)** | **Axilla Level I (posterior)** | **Axilla Level I (inter-pectoral)** | **Axilla Level II** | **Axilla Level III** | **Internal Mammary** | **Supra-clavicular** | **Mediastinal** | **Interval** | **Contra-lateral** |
| **0 (central)** | 78.4 (69.8, 85.1) | 33.3 (25.2, 42.6) | 0.9 (0.1, 6.1) | 8.1 (4.3, 14.9) | 4.5 (1.9, 10.4) | 12.6 (7.6, 20.2) | 0.9 (0.1, 6.1) | 33.3 (25.2, 42.6) | 0.9 (0.1, 6.1) | 0.9 (0.1, 6.1) | 11.7 (6.9, 19.1) | 0.9 (0.1, 6.1) |
| **1** | 77.6 (67.6, 85.3) | 22.4 (14.7, 32.4) | 3.5 (1.1, 10.4) | 7.1 (3.2, 14.8) | 3.5 (1.1, 10.4) | 10.6 (5.6, 19.1) | 0.0 (0.0, nan) | 22.4 (14.7, 32.4) | 1.2 (0.2, 7.9) | 0.0 (0.0, nan) | 4.7 (1.8, 11.9) | 0.0 (0.0, nan) |
| **2** | 82.4 (76.5, 87.1) | 29.6 (23.7, 36.4) | 1.0 (0.3, 3.9) | 11.1 (7.4, 16.2) | 2.5 (1.0, 5.9) | 6.5 (3.8, 10.9) | 0.5 (0.1, 3.5) | 14.1 (9.9, 19.6) | 0.5 (0.1, 3.5) | 0.0 (0.0, nan) | 8.0 (5.0, 12.7) | 0.0 (0.0, nan) |
| **3 (lateral)** | 73.4 (63.6, 81.3) | 30.9 (22.4, 40.9) | 1.1 (0.1, 7.2) | 12.8 (7.4, 21.1) | 2.1 (0.5, 8.1) | 7.4 (3.6, 14.8) | 1.1 (0.1, 7.2) | 14.9 (9.0, 23.6) | 0.0 (0.0, nan) | 0.0 (0.0, nan) | 11.7 (6.6, 19.9) | 0.0 (0.0, nan) |
| **4** | 78.6 (65.9, 87.4) | 32.1 (21.3, 45.4) | 0.0 (0.0, nan) | 12.5 (6.1, 24.0) | 1.8 (0.3, 11.6) | 5.4 (1.7, 15.3) | 0.0 (0.0, nan) | 28.6 (18.3, 41.7) | 0.0 (0.0, nan) | 0.0 (0.0, nan) | 7.1 (2.7, 17.5) | 0.0 (0.0, nan) |
| **5** | 75.6 (60.3, 86.3) | 26.8 (15.5, 42.3) | 0.0 (0.0, nan) | 17.1 (8.4, 31.7) | 4.9 (1.2, 17.5) | 7.3 (2.4, 20.4) | 0.0 (0.0, nan) | 39.0 (25.5, 54.5) | 0.0 (0.0, nan) | 0.0 (0.0, nan) | 24.4 (13.7, 39.7) | 0.0 (0.0, nan) |
| **6 (inferior)** | 64.5 (46.6, 79.1) | 32.3 (18.3, 50.3) | 3.2 (0.5, 19.6) | 16.1 (6.9, 33.4) | 0.0 (0.0, nan) | 0.0 (0.0, nan) | 0.0 (0.0, nan) | 51.6 (34.5, 68.3) | 0.0 (0.0, nan) | 3.2 (0.5, 19.6) | 16.1 (6.9, 33.4) | 0.0 (0.0, nan) |
| **7** | 87.5 (67.6, 95.9) | 20.8 (8.9, 41.3) | 0.0 (0.0, nan) | 4.2 (0.6, 24.4) | 0.0 (0.0, nan) | 8.3 (2.1, 27.9) | 0.0 (0.0, nan) | 70.8 (50.2, 85.4) | 0.0 (0.0, nan) | 0.0 (0.0, nan) | 16.7 (6.4, 36.9) | 0.0 (0.0, nan) |
| **8** | 82.6 (61.8, 93.3) | 30.4 (15.3, 51.5) | 0.0 (0.0, nan) | 8.7 (2.2, 28.9) | 0.0 (0.0, nan) | 13.0 (4.3, 33.5) | 0.0 (0.0, nan) | 65.2 (44.3, 81.6) | 0.0 (0.0, nan) | 0.0 (0.0, nan) | 4.3 (0.6, 25.2) | 0.0 (0.0, nan) |
| **9 (medial)** | 82.6 (61.8, 93.3) | 26.1 (12.2, 47.2) | 0.0 (0.0, nan) | 0.0 (0.0, nan) | 0.0 (0.0, nan) | 4.3 (0.6, 25.2) | 0.0 (0.0, nan) | 47.8 (28.8, 67.5) | 0.0 (0.0, nan) | 4.3 (0.6, 25.2) | 4.3 (0.6, 25.2) | 0.0 (0.0, nan) |
| **10** | 75.0 (62.1, 84.6) | 28.6 (18.3, 41.7) | 1.8 (0.3, 11.6) | 5.4 (1.7, 15.3) | 0.0 (0.0, nan) | 8.9 (3.8, 19.7) | 0.0 (0.0, nan) | 50.0 (37.2, 62.8) | 1.8 (0.3, 11.6) | 0.0 (0.0, nan) | 5.4 (1.7, 15.3) | 1.8 (0.3, 11.6) |
| **11** | 68.3 (55.6, 78.8) | 23.3 (14.3, 35.6) | 1.7 (0.2, 10.9) | 11.7 (5.7, 22.5) | 1.7 (0.2, 10.9) | 6.7 (2.5, 16.5) | 0.0 (0.0, nan) | 48.3 (36.1, 60.8) | 1.7 (0.2, 10.9) | 1.7 (0.2, 10.9) | 10.0 (4.6, 20.5) | 0.0 (0.0, nan) |
| **12 (superior)** | 72.7 (60.8, 82.1) | 33.3 (23.1, 45.5) | 1.5 (0.2, 10.0) | 6.1 (2.3, 15.1) | 3.0 (0.8, 11.3) | 12.1 (6.2, 22.4) | 1.5 (0.2, 10.0) | 27.3 (17.9, 39.2) | 0.0 (0.0, nan) | 0.0 (0.0, nan) | 7.6 (3.2, 16.9) | 0.0 (0.0, nan) |
| **All** | 77.2 (74.3, 79.9) | 29.1 (26.2, 32.2) | 1.3 (0.7, 2.3) | 9.8 (8.0, 11.9) | 2.4 (1.6, 3.7) | 8.3 (6.6, 10.3) | 0.5 (0.2, 1.2) | 30.4 (27.4, 33.5) | 0.6 (0.2, 1.4) | 0.5 (0.2, 1.2) | 9.6 (7.8, 11.7) | 0.2 (0.1, 0.9) |

## Tumour Prevalence

**Table S5** Bayesian tumour prevalence to each tumour region from each node field, reported as mean % (95% CI).

| Node Field | Tumour Region (L breast) | | | | | | | | | | | | |
|---|---|---|---|---|---|---|---|---|---|---|---|---|---|
| | **0 (central)** | **1** | **2** | **3 (lateral)** | **4** | **5** | **6 (inferior)** | **7** | **8** | **9 (medial)** | **10** | **11** | **12 (superior)** |
| **Axilla Level I (anterior)** | 12.9 (10.5, 15.5) | 9.8 (7.7, 12.1) | 24.1 (21.0, 27.4) | 10.2 (8.1, 12.6) | 6.6 (4.8, 8.6) | 4.7 (3.2, 6.4) | 3.1 (1.9, 4.5) | 3.2 (2.0, 4.7) | 2.9 (1.8, 4.3) | 2.9 (1.8, 4.3) | 6.3 (4.6, 8.2) | 6.1 (4.5, 8.1) | 7.2 (5.4, 9.2) |
| **Axilla Level I (medial)** | 14.3 (10.4, 18.7) | 7.5 (4.7, 11.0) | 22.6 (17.7, 27.8) | 11.3 (7.8, 15.3) | 7.1 (4.4, 10.5) | 4.5 (2.4, 7.3) | 4.1 (2.1, 6.8) | 2.3 (0.8, 4.3) | 3.0 (1.3, 5.4) | 2.6 (1.1, 4.9) | 6.4 (3.8, 9.6) | 5.6 (3.2, 8.7) | 8.6 (5.6, 12.3) |
| **Axilla Level I (lateral)** | 8.3 (1.1, 21.9) | 16.7 (5.0, 33.6) | 12.5 (2.8, 28.0) | 8.3 (1.1, 22.0) | 4.2 (0.1, 14.8) | 4.2 (0.1, 14.8) | 8.3 (1.1, 21.9) | 4.2 (0.1, 14.8) | 4.2 (0.1, 14.8) | 4.2 (0.1, 14.8) | 8.3 (1.1, 22.0) | 8.3 (1.1, 21.9) | 8.3 (1.1, 21.9) |
| **Axilla Level I (posterior)** | 10.2 (5.1, 16.9) | 7.1 (3.0, 13.0) | 23.5 (15.7, 32.3) | 13.3 (7.3, 20.6) | 8.2 (3.6, 14.3) | 8.2 (3.6, 14.3) | 6.1 (2.3, 11.6) | 2.0 (0.3, 5.6) | 3.1 (0.6, 7.3) | 1.0 (0.0, 3.7) | 4.1 (1.1, 8.8) | 8.2 (3.6, 14.3) | 5.1 (1.7, 10.2) |
| **Axilla Level I (inter-pectoral)** | 17.6 (7.0, 31.9) | 11.8 (3.4, 24.3) | 17.6 (7.0, 31.9) | 8.8 (1.9, 20.2) | 5.9 (0.7, 15.8) | 8.8 (1.9, 20.2) | 2.9 (0.1, 10.6) | 2.9 (0.1, 10.6) | 2.9 (0.1, 10.5) | 2.9 (0.1, 10.6) | 2.9 (0.1, 10.6) | 5.9 (0.7, 15.8) | 8.8 (1.9, 20.3) |
| **Axilla Level II** | 17.6 (10.4, 26.4) | 11.8 (5.8, 19.3) | 16.5 (9.4, 25.0) | 9.4 (4.2, 16.4) | 4.7 (1.3, 10.1) | 4.7 (1.3, 10.1) | 1.2 (0.0, 4.3) | 3.5 (0.7, 8.3) | 4.7 (1.3, 10.1) | 2.4 (0.3, 6.5) | 7.1 (2.7, 13.3) | 5.9 (2.0, 11.8) | 10.6 (5.0, 17.9) |
| **Axilla Level III** | 11.8 (1.5, 30.2) | 5.9 (0.2, 20.7) | 11.8 (1.5, 30.2) | 11.8 (1.6, 30.2) | 5.9 (0.2, 20.5) | 5.9 (0.2, 20.6) | 5.9 (0.2, 20.6) | 5.9 (0.2, 20.6) | 5.9 (0.2, 20.6) | 5.9 (0.2, 20.6) | 5.9 (0.2, 20.6) | 5.9 (0.2, 20.6) | 11.8 (1.6, 30.2) |
| **Internal Mammary** | 13.7 (9.9, 18.0) | 7.2 (4.5, 10.5) | 10.5 (7.1, 14.3) | 5.4 (3.1, 8.4) | 6.1 (3.6, 9.2) | 6.1 (3.6, 9.2) | 6.1 (3.6, 9.2) | 6.5 (3.9, 9.7) | 5.8 (3.3, 8.8) | 4.3 (2.3, 7.0) | 10.5 (7.1, 14.3) | 10.8 (7.5, 14.7) | 6.9 (4.2, 10.1) |
| **Supra-clavicular** | 11.1 (1.5, 28.7) | 11.1 (1.5, 28.6) | 11.1 (1.5, 28.6) | 5.6 (0.2, 19.6) | 5.5 (0.1, 19.5) | 5.6 (0.1, 19.5) | 5.5 (0.1, 19.5) | 5.6 (0.1, 19.5) | 5.5 (0.1, 19.4) | 5.6 (0.1, 19.5) | 11.1 (1.5, 28.7) | 11.1 (1.5, 28.7) | 5.6 (0.1, 19.6) |
| **Media-stinal** | 11.8 (1.5, 30.3) | 5.9 (0.2, 20.6) | 5.9 (0.2, 20.6) | 5.9 (0.2, 20.6) | 5.9 (0.2, 20.7) | 5.9 (0.2, 20.6) | 11.8 (1.6, 30.3) | 5.9 (0.2, 20.6) | 5.9 (0.2, 20.6) | 11.8 (1.6, 30.2) | 5.9 (0.2, 20.5) | 11.8 (1.6, 30.3) | 5.9 (0.2, 20.6) |
| **Interval** | 14.6 (8.3, 22.2) | 5.2 (1.7, 10.4) | 17.7 (10.8, 25.9) | 12.5 (6.7, 19.8) | 5.2 (1.7, 10.4) | 11.5 (5.9, 18.5) | 6.3 (2.4, 11.9) | 5.2 (1.7, 10.4) | 2.1 (0.3, 5.7) | 2.1 (0.3, 5.7) | 4.2 (1.2, 9.0) | 7.3 (3.0, 13.2) | 6.2 (2.4, 11.9) |
| **Contra-lateral** | 13.3 (1.8, 33.9) | 6.7 (0.2, 23.2) | 6.7 (0.2, 23.1) | 6.7 (0.2, 23.2) | 6.7 (0.2, 23.2) | 6.7 (0.2, 23.1) | 6.7 (0.2, 23.2) | 6.7 (0.2, 23.2) | 6.7 (0.2, 23.2) | 6.7 (0.2, 23.2) | 13.3 (1.8, 33.9) | 6.7 (0.2, 23.2) | 6.7 (0.2, 23.2) |
| **All** | 12.7 (10.6, 15.0) | 9.7 (7.9, 11.8) | 22.7 (20.0, 25.5) | 10.8 (8.8, 12.9) | 6.5 (4.9, 8.2) | 4.8 (3.5, 6.3) | 3.6 (2.5, 5.0) | 2.8 (1.8, 4.0) | 2.7 (1.8, 3.9) | 2.7 (1.8, 3.9) | 6.5 (4.9, 8.2) | 6.9 (5.3, 8.7) | 7.6 (5.9, 9.4) |

**Table S6** Bootstrap tumour prevalence to each tumour region from each node field, reported as mean % (95% CI)

| Node Field | Tumour Region (L breast) | | | | | | | | | | | | |
|---|---|---|---|---|---|---|---|---|---|---|---|---|---|
| | **0 (central)** | **1** | **2** | **3 (lateral)** | **4** | **5** | **6 (inferior)** | **7** | **8** | **9 (medial)** | **10** | **11** | **12 (superior)** |
| **Axilla Level I (anterior)** | 13.0 (10.4, 15.5) | 9.8 (7.6, 12.1) | 24.4 (21.2, 27.7) | 10.3 (8.0, 12.7) | 6.6 (4.8, 8.5) | 4.6 (3.1, 6.3) | 3.0 (1.8, 4.3) | 3.1 (1.9, 4.5) | 2.8 (1.6, 4.2) | 2.8 (1.6, 4.2) | 6.3 (4.5, 8.2) | 6.1 (4.3, 8.0) | 7.2 (5.2, 9.1) |
| **Axilla Level I (medial)** | 14.6 (10.3, 19.0) | 7.5 (4.3, 11.1) | 23.3 (18.2, 28.9) | 11.5 (7.9, 15.4) | 7.1 (4.0, 10.3) | 4.3 (2.0, 7.1) | 4.0 (1.6, 6.3) | 2.0 (0.4, 4.0) | 2.8 (0.8, 5.1) | 2.4 (0.8, 4.3) | 6.3 (3.6, 9.5) | 5.5 (2.8, 8.7) | 8.7 (5.5, 12.3) |
| **Axilla Level I (lateral)** | 9.1 (0, 27.3) | 27.2 (0, 54.5) | 18.2 (0, 45.5) | 9.1 (0, 27.3) | 0.0 (0, 0.0) | 0.0 (0, 0.0) | 9.1 (0, 27.3) | 0.0 (0, 0.0) | 0.0 (0, 0.0) | 0.0 (0, 0.0) | 9.1 (0, 27.3) | 9.1 (0, 27.3) | 9.1 (0, 27.3) |
| **Axilla Level I (posterior)** | 10.6 (4.7, 17.6) | 7.1 (2.4, 12.9) | 25.9 (16.5, 35.3) | 14.1 (7.1, 22.4) | 8.2 (3.5, 14.1) | 8.3 (3.5, 14.1) | 5.9 (1.2, 11.8) | 1.2 (0.0, 3.5) | 2.4 (0.0, 5.9) | 0.0 (0.0, 0.0) | 3.5 (0.0, 8.2) | 8.2 (2.4, 14.1) | 4.7 (1.2, 9.4) |
| **Axilla Level I (inter-pectoral)** | 23.8 (4.8, 42.9) | 14.3 (0.0, 28.6) | 23.8 (4.8, 42.9) | 9.5 (0.0, 23.8) | 4.8 (0.0, 14.3) | 9.6 (0.0, 23.8) | 0.0 (0.0, 0.0) | 0.0 (0.0, 0.0) | 0.0 (0.0, 0.0) | 0.0 (0.0, 0.0) | 0.0 (0.0, 0.0) | 4.7 (0.0, 14.3) | 9.5 (0.0, 23.8) |
| **Axilla Level II** | 19.4 (11.1, 29.2) | 12.5 (5.6, 20.8) | 18.1 (9.7, 27.8) | 9.7 (4.2, 16.7) | 4.2 (0.0, 9.7) | 4.2 (0.0, 9.7) | 0.0 (0.0, 0.0) | 2.8 (0.0, 6.9) | 4.2 (0.0, 9.7) | 1.4 (0.0, 4.2) | 7.0 (1.4, 13.9) | 5.5 (1.4, 11.1) | 11.1 (4.2, 19.4) |
| **Axilla Level III** | 25.2 (0, 75) | 0.0 (0, 0) | 24.9 (0, 75) | 25.0 (0, 75) | 0.0 (0, 0) | 0.0 (0, 0) | 0.0 (0, 0) | 0.0 (0, 0) | 0.0 (0, 0) | 0.0 (0, 0) | 0.0 (0, 0) | 0.0 (0, 0) | 24.8 (0, 75) |
| **Internal Mammary** | 14.0 (9.8, 18.2) | 7.2 (4.2, 10.6) | 10.6 (7.2, 14.4) | 5.3 (2.7, 8.3) | 6.1 (3.4, 9.1) | 6.1 (3.4, 9.1) | 6.1 (3.4, 9.1) | 6.4 (3.8, 9.5) | 5.7 (3.0, 8.7) | 4.2 (1.9, 6.8) | 10.6 (7.2, 14.4) | 11.0 (7.2, 14.8) | 6.8 (3.8, 9.8) |
| **Supra-clavicular** | 20.1 (0, 60) | 20.1 (0, 60) | 20.0 (0, 60) | 0.0 (0, 0) | 0.0 (0, 0) | 0.0 (0, 0) | 0.0 (0, 0) | 0.0 (0, 0) | 0.0 (0, 0) | 0.0 (0, 0) | 19.9 (0, 60) | 20.1 (0, 60) | 0.0 (0, 0) |
| **Media-stinal** | 25.1 (0, 75) | 0.0 (0, 0) | 0.0 (0, 0) | 0.0 (0, 0) | 0.0 (0, 0) | 0.0 (0, 0) | 25.0 (0, 75) | 0.0 (0, 0) | 0.0 (0, 0) | 25.0 (0, 75) | 0.0 (0, 0) | 24.9 (0, 75) | 0.0 (0, 0) |
| **Interval** | 15.7 (8.4, 24.1) | 4.8 (1.2, 9.6) | 19.3 (10.8, 27.7) | 13.2 (6.0, 20.5) | 4.8 (1.2, 9.6) | 12.1 (6.0, 19.3) | 6.0 (1.2, 12.0) | 4.8 (1.2, 9.6) | 1.2 (0.0, 3.6) | 1.2 (0.0, 3.6) | 3.6 (0.0, 8.4) | 7.2 (2.4, 13.3) | 6.0 (1.2, 12.0) |
| **Contra-lateral** | 50.1 (0, 100) | 0.0 (0, 0) | 0.0 (0, 0) | 0.0 (0, 0) | 0.0 (0, 0) | 0.0 (0, 0) | 0.0 (0, 0) | 0.0 (0, 0) | 0.0 (0, 0) | 0.0 (0, 0) | 49.9 (0, 100) | 0.0 (0, 0) | 0.0 (0, 0) |
| **All** | 12.8 (10.6, 15.1) | 9.8 (7.8, 11.7) | 22.9 (20.1, 25.8) | 10.8 (8.7, 12.9) | 6.4 (4.8, 8.2) | 4.7 (3.3, 6.2) | 3.6 (2.4, 4.8) | 2.8 (1.7, 3.9) | 2.6 (1.6, 3.8) | 2.6 (1.6, 3.8) | 6.4 (4.8, 8.2) | 6.9 (5.3, 8.6) | 7.6 (5.9, 9.4) |

**Table S7** Regression tumour prevalence to each tumour region from each node field, reported as mean % (95% CI)

| Node Field | Tumour Region (L breast) | | | | | | | | | | | | |
|---|---|---|---|---|---|---|---|---|---|---|---|---|---|
| | **0 (central)** | **1** | **2** | **3 (lateral)** | **4** | **5** | **6 (inferior)** | **7** | **8** | **9 (medial)** | **10** | **11** | **12 (superior)** |
| **Axilla Level I (anterior)** | 13.0 (9.7, 16.5) | 9.8 (6.6, 13.3) | 24.4 (21.2, 27.9) | 10.3 (7.0, 13.8) | 6.6 (3.3, 10.1) | 4.6 (1.3, 8.1) | 3.0 (0.0, 6.5) | 3.1 (0.0, 6.6) | 2.8 (0.0, 6.3) | 2.8 (0.0, 6.3) | 6.3 (3.0, 9.8) | 6.1 (2.8, 9.6) | 7.2 (3.9, 10.7) |
| **Axilla Level I (medial)** | 14.6 (9.5, 20.4) | 7.5 (2.4, 13.3) | 23.3 (18.2, 29.1) | 11.5 (6.3, 17.3) | 7.1 (2.0, 12.9) | 4.3 (0.0, 10.2) | 4.0 (0.0, 9.8) | 2.0 (0.0, 7.8) | 2.8 (0.0, 8.6) | 2.4 (0.0, 8.2) | 6.3 (1.2, 12.1) | 5.5 (0.4, 11.4) | 8.7 (3.6, 14.5) |
| **Axilla Level I (lateral)** | 9.1 (0.0, 42.3) | 27.3 (9.1, 60.5) | 18.2 (0.0, 51.4) | 9.1 (0.0, 42.3) | 0.0 (0.0, 33.2) | 0.0 (0.0, 33.2) | 9.1 (0.0, 42.3) | 0.0 (0.0, 33.2) | 0.0 (0.0, 33.2) | 0.0 (0.0, 33.2) | 9.1 (0.0, 42.3) | 9.1 (0.0, 42.3) | 9.1 (0.0, 42.3) |
| **Axilla Level I (posterior)** | 10.6 (2.4, 21.1) | 7.1 (0.0, 17.6) | 25.9 (17.6, 36.4) | 14.1 (5.9, 24.6) | 8.2 (0.0, 18.8) | 8.2 (0.0, 18.8) | 5.9 (0.0, 16.4) | 1.2 (0.0, 11.7) | 2.4 (0.0, 12.9) | 0.0 (0.0, 10.5) | 3.5 (0.0, 14.1) | 8.2 (0.0, 18.8) | 4.7 (0.0, 15.2) |
| **Axilla Level I (inter-pectoral)** | 23.8 (9.5, 47.3) | 14.3 (0.0, 37.8) | 23.8 (9.5, 47.3) | 9.5 (0.0, 33.0) | 4.8 (0.0, 28.3) | 9.5 (0.0, 33.0) | 0.0 (0.0, 23.5) | 0.0 (0.0, 23.5) | 0.0 (0.0, 23.5) | 0.0 (0.0, 23.5) | 0.0 (0.0, 23.5) | 4.8 (0.0, 28.3) | 9.5 (0.0, 33.0) |
| **Axilla Level II** | 19.4 (9.7, 30.0) | 12.5 (2.8, 23.0) | 18.1 (8.3, 28.6) | 9.7 (0.0, 20.3) | 4.2 (0.0, 14.7) | 4.2 (0.0, 14.7) | 0.0 (0.0, 10.5) | 2.8 (0.0, 13.3) | 4.2 (0.0, 14.7) | 1.4 (0.0, 11.9) | 6.9 (0.0, 17.5) | 5.6 (0.0, 16.1) | 11.1 (1.4, 21.6) |
| **Axilla Level III** | 25 (0, 86.9) | 0 (0, 61.9) | 25 (0, 86.9) | 25 (0, 86.9) | 0 (0, 61.9) | 0 (0, 61.9) | 0 (0, 61.9) | 0 (0, 61.9) | 0 (0, 61.9) | 0 (0, 61.9) | 0 (0, 61.9) | 0 (0, 61.9) | 25 (0, 86.9) |
| **Internal Mammary** | 14.0 (9.5, 19.2) | 7.2 (2.7, 12.4) | 10.6 (6.1, 15.8) | 5.3 (0.8, 10.5) | 6.1 (1.5, 11.2) | 6.1 (1.5, 11.2) | 6.1 (1.5, 11.2) | 6.4 (1.9, 11.6) | 5.7 (1.1, 10.8) | 4.2 (0.0, 9.3) | 10.6 (6.1, 15.8) | 11.0 (6.4, 16.2) | 6.8 (2.3, 12.0) |
| **Supra-clavicular** | 20 (0, 75.3) | 20 (0, 75.3) | 20 (0, 75.3) | 0 (0, 55.3) | 0 (0, 55.3) | 0 (0, 55.3) | 0 (0, 55.3) | 0 (0, 55.3) | 0 (0, 55.3) | 0 (0, 55.3) | 20 (0, 75.3) | 20 (0, 75.3) | 0 (0, 55.3) |
| **Media-stinal** | 25 (0, 86.9) | 0 (0, 61.9) | 0 (0, 61.9) | 0 (0, 61.9) | 0 (0, 61.9) | 0 (0, 61.9) | 25 (0, 86.9) | 0 (0, 61.9) | 0 (0, 61.9) | 25 (0, 86.9) | 0 (0, 61.9) | 25 (0, 86.9) | 0 (0, 61.9) |
| **Interval** | 15.7 (7.2, 25.9) | 4.8 (0.0, 15.0) | 19.3 (10.8, 29.5) | 13.3 (4.8, 23.5) | 4.8 (0.0, 15.0) | 12.0 (3.6, 22.3) | 6.0 (0.0, 16.3) | 4.8 (0.0, 15.0) | 1.2 (0.0, 11.4) | 1.2 (0.0, 11.4) | 3.6 (0.0, 13.8) | 7.2 (0.0, 17.5) | 6.0 (0.0, 16.3) |
| **Contra-lateral** | 50 (50, 100.0) | 0 (0, 95.8) | 0 (0, 95.8) | 0 (0, 95.8) | 0 (0, 95.8) | 0 (0, 95.8) | 0 (0, 95.8) | 0 (0, 95.8) | 0 (0, 95.8) | 0 (0, 95.8) | 50 (50, 100.0) | 0 (0, 95.8) | 0 (0, 95.8) |
| **All** | 12.8 (9.9, 15.8) | 9.8 (6.9, 12.8) | 22.9 (20.0, 25.9) | 10.8 (7.9, 13.9) | 6.4 (3.6, 9.5) | 4.7 (1.8, 7.8) | 3.6 (0.7, 6.6) | 2.8 (0.0, 5.8) | 2.6 (0.0, 5.7) | 2.6 (0.0, 5.7) | 6.4 (3.6, 9.5) | 6.9 (4.0, 9.9) | 7.6 (4.7, 10.6) |

## Registration Validation

**Table S8** The mean registration errors and inter-observer variations for each landmark

| Landmark | Mean Registration Error (mm) | | Mean Inter-observer Variation (mm) | | |
|---|---|---|---|---|---|
| | **Linear Registration** | **Deformable Registration** | **Observers 1 and 2** | **Observers 1 and 3** | **Observers 2 and 3** |
| left scapula | 144.4 | 7.9 | 11.8 | 8.1 | 8.6 |
| right scapula | 143.1 | 13.0 | 10.6 | 8.2 | 5.7 |
| left humerus | 142.6 | 11.2 | 9.6 | 4.9 | 12.9 |
| right humerus | 144.2 | 6.1 | 6.7 | 3.7 | 7.6 |
| left clavicle | 142.3 | 5.8 | 7.2 | 6.4 | 6.2 |
| right clavicle | 141.8 | 6.1 | 5.2 | 5.1 | 4.5 |
| sternum | 138.9 | 6.0 | 5.2 | 5.7 | 2.4 |
| left rib 2 | 137.8 | 10.8 | 4.8 | 10.1 | 7.5 |
| right rib 2 | 137.2 | 11.3 | 4.9 | 9.8 | 9.0 |
| left rib 3 | 128.1 | 14.3 | 3.9 | 7.3 | 9.7 |
| right rib 3 | 132.3 | 12.1 | 3.5 | 9.3 | 10.1 |
| left inferior scapula | 137.8 | 19.6 | 4.5 | 5.9 | 4.3 |
| right inferior scapula | 133.3 | 20.7 | 7.8 | 5.9 | 7.5 |
| **Mean (SD)** | **138.7 (5.1)** | **11.2 (4.9)** | **6.6 (2.7)** | **6.9 (2.0)** | **7.4 (2.8)** |
